# Diverse regulatory factors associate with flowering time and yield responses in winter-type *Brassica napus*

**DOI:** 10.1186/s12864-015-1950-1

**Published:** 2015-09-29

**Authors:** Sarah Schiessl, Federico Iniguez-Luy, Wei Qian, Rod J. Snowdon

**Affiliations:** Department of Plant Breeding, Justus Liebig University, IFZ Research Centre for Biosystems, Land Use and Nutrition, Heinrich-Buff-Ring 26-32, 35392 Giessen, Germany; Agriaquaculture Nutritional Genomic Center (CGNA), Genomics and Bioinformatics Unit, Km 10 Camino Cajón-Vilcún, INIA, Temuco, Chile; College of Agronomy and Biotechnology, Southwest University, 400716 Chongqing, China

**Keywords:** GWAS, Rapeseed, Population structure, Post-transcriptional regulation, SNP marker, Gene ontology enrichment

## Abstract

**Background:**

Flowering time, plant height and seed yield are strongly influenced by climatic and day-length adaptation in crop plants. To investigate these traits under highly diverse field conditions in the important oilseed crop *Brassica napus*, we performed a genome-wide association study using data from diverse agroecological environments spanning three continents.

**Methods:**

A total of 158 European winter-type *B.napus* inbred lines were genotyped with 21,623 unique, single-locus single-nucleotide polymorphism (SNP) markers using the Brassica 60 K-SNP Illumina® Infinium consortium array. Phenotypic associations were calculated in the panel over the years 2010–2012 for flowering time, plant height and seed yield in 5 highly diverse locations in Germany, China and Chile, adding up to 11 diverse environments in total.

**Results:**

We identified 101 genome regions associating with the onset of flowering, 69 with plant height, 36 with seed yield and 68 cross-trait regions with potential adaptive value. Within these regions, *B.napus* orthologs for a number of candidate adaptation genes were detected, including central circadian clock components like *CIRCADIAN CLOCK- ASSOCIATED 1* (*Bna.CCA1*) and the important flowering-time regulators *FLOWERING LOCUS T* (*Bna.FT*) and *FRUITFUL* (*Bna.FUL*).

**Discussion:**

Gene ontology (GO) enrichment analysis of candidate regions suggested that selection of genes involved in post-transcriptional and epigenetic regulation of flowering time may play a potential role in adaptation of *B. napus* to highly divergent environments. The classical flowering time regulators *Bna.FLC* and *Bna.CO* were not found among the candidate regions, although both show functional variation. Allelic effects were additive for plant height and yield, but not for flowering time. The scarcity of positive minor alleles for yield in this breeding pool points to a lack of diversity for adaptation that could restrict yield gain in the face of environmental change.

**Conclusions:**

Our study provides a valuable framework to further improve the adaptability and yield stability of this recent allopolyploid crop under changing environments. The results suggest that flowering time regulation within an adapted *B. napus* breeding pool is driven by a high number of small modulating processes rather than major transcription factors like *Bna.CO*. In contrast, yield regulation appears highly parallel, therefore yield could be increased by pyramiding positively associated haplotypes.

**Electronic supplementary material:**

The online version of this article (doi:10.1186/s12864-015-1950-1) contains supplementary material, which is available to authorized users.

## Background

Environmental adaptation is decisive for optimal yield of major crop plants. This includes adaptation of the reproductive system to the prevailing climatic conditions (i.e., season, day length, and temperature) and an appropriate response to biotic and abiotic stresses. Adaptation of the reproductive system is mainly achieved by setting the time for flowering, which is considered to be the transition from the vegetative to the generative phase of the plant lifecycle. This switch determines how much the plant invests into vegetative growth and, consequently, how many nutrients will be available for remobilization at the time of seed filling [1]. This control occurs both directly, depending on the leaf area and/or the root architecture, and indirectly, via synchronization with day length, water availability and ambient temperature. In temperate regions, flowering time also determines the time left for seed maturation before winter. The complex task of finding the optimal time to switch is controlled by the flowering gene network, representing a set of interacting pathways of transcription factors, photosensors, enzymes and miRNAs (reviewed in depth in [2–8]).

In *Arabidopsis thaliana* it has been shown before that optimal flowering time is correlated with higher seed yield [9]. A recent study in *Brassica rapa* identified *B.rapa FLOWERING LOCUS C* (*Bra.FLC*) as a candidate for a quantitative trait locus (QTL) contributing to transgenerative fitness, i.e., the influence of flowering time of the mother plant on germination of the offspring [10]. In *Brassica napus*, earlier QTL studies showed colocalization of QTL for flowering and traits like yield and plant height [11–13], suggesting that QTL-associated flowering genes like *B.napus FLOWERING LOCUS C* (*Bna.FLC*), *FLOWERING LOCUS T* (*Bna.FT*), *VERNALISATION INSENSITIVE 3* (*Bna.VIN3*) and *ZEITLUPE* (*Bna.ZTL*) have a major impact on these traits. This is indicative for a more general role of flowering regulators in plant development, making them global regulators of plant adaptation. Such a role has been demonstrated in *A.thaliana* for *Ath.FLC* [14], *CRYPTOCHROME 2* (*Ath.CRY2*), *FRIGIDA* (*Ath.FRI*) and *PHYTOCHROME C* (*Ath.PHYC*) [1].

*Brassica napus* (rapeseed, oilseed rape, canola) is a recent allotetraploid crop species with high economic impact in Europe, Asia, Australia and North America [15]. Its huge phenotypic variation groups the species into different morphotypes with distinct flowering behavior, vernalisation requirement and winter hardiness. Being closely related to the model crucifer *A.thaliana*, *B.napus* serves as appropriate model for effects of allopolyploidy [16–19]. It harbors two related subgenomes from a recent interspecific hybridization (A subgenome with 10 chromosomes from *B.rapa* and C subgenome with 9 chromosomes from *B. oleracea*). Polyploidy, although widely spread among crop species, poses special challenges for molecular breeding, as the similarity of subgenomes can impede development of locus-specific markers, cloning of candidate genes or knock-out techniques. Recently developed resources like the Brassica 60 k SNP Illumina® Infinium consortium array and the publication of the *Brassica napus* genomic sequence [20] now allow fine-mapping of genotype-trait associations on a genome-wide scale.

The genetics of flowering, plant development and yield have been studied extensively in *Brassica* crop species by QTL analysis in biparental populations [11–13, 21–26] and association genetics [27–29]. Biparental QTL studies generally suffer from their low resolution and only assess alleles present in the mapping parents; therefore the results may not be applicable within or across breeding pools. Genome-wide association studies overcome these restrictions, given reasonable marker saturation and an appropriately diverse population. Cai and co-authors [27] used 1191 markers to study 192 inbred lines to map yield-related traits, whereas Li and co-authors [28] increased the number of markers almost 20-fold by using the Brassica 60 k SNP array to study seed weight and quality traits in 472 diverse *B.napus* lines. High-resolution identification of the well-known *FATTY ACID ELONGASE 1* (*Bna.FAE1*) homologs controlling erucic acid content demonstrated the power of the GWAS approach to map key genes for important traits using densely-spaced genome-wide SNP markers.

Genes for global plant adaptation are highly interesting markers for plant breeding. Biparental QTL studies have demonstrated that variants of the major flowering-time regulatory genes like *FLOWERING LOCUS C* (*Bna.FLC*) and *FLOWERING LOCUS T* (*Bna.FT*) are the major causes for the broad ecogeographical diversification within *B.napus* into spring-type, winter-type and semi-winter rapeseed gene pools [12, 22, 26, 30]. Because of the extremely strong phenotypic effects of these few loci, in across-pool populations the genetic effects of loci with regulatory roles within each morphotype tend to be masked, making it difficult to reveal subtle effects of floral regulators on local adaptation. These regulators are often important for breeding because they allow breeders to adjust yield-related traits within adapted gene pools. Association mapping within pools can potentially reveal allelic variation in regulatory loci responsible for local adaptability, for example to environmental variation or climate change.

To reveal if selected members of the flowering network are valuable regulatory factors of plant adaptability, we phenotyped a set of 158 diverse winter-type *B.napus* inbred lines in extremely different environments in three location in Germany (2010–2012), and one location in Chile (2011–2012) and China (2012). Phenotypic data was collected for onset of flowering, plant height and yield and used to calculate genome-wide associations with genotype data from the Brassica 60 k SNP array. By investigating the gene content and annotations within chromosome regions in linkage disequilibrium (LD) with QTL for these three traits, we aimed to gain insight into the mechanisms of adaptation to fluctuations in photoperiodicity and climatic fluctuation in this important global crop.

## Results

### Field trials

**Fig. 1:**
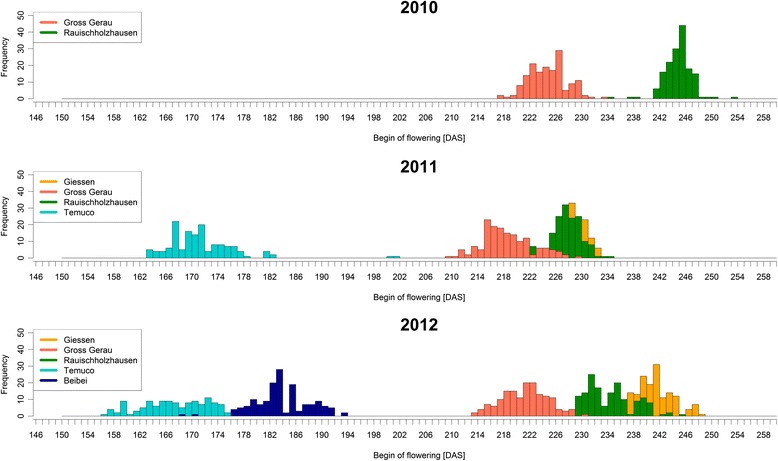
Distribution of flowering time. Values in Days after sowing (DAS) for all 11 environments in 3 years (2010–2012) and 5 locations (Germany: Giessen, Gross Gerau, Rauischholzhausen; Chile: Temuco; China: Beibei, Chongqing). The onset of flowering was attributed when 10 % of the plants in a plot reached BBCH stage 61

**Fig. 2:**
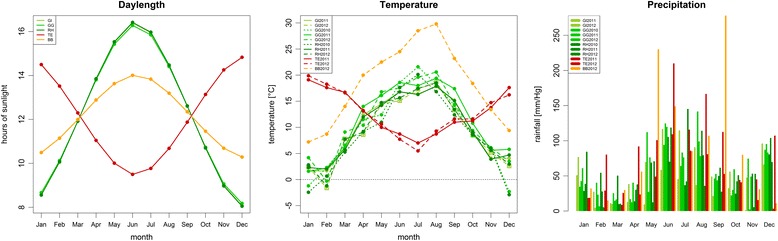
Climate data for all environments tested. The graphs show mean values (daylength, temperature) and monthly sums (precipitation) for each location (daylength) and each environment (temperature, precipitation). The environments are abbreviated as follows: Germany: Giessen (GI), Gross Gerau (GG), Rauischholzhausen (RH); Chile: Temuco (TE); China: Beibei, Chongqing (BB), with their respective year of harvest. For precipitation, consecutive years are placed consecutively beneath each other

The beginning of flowering (BOF) varied considerably among years and locations, spanning over a range of 97 days from 157 days after sowing (DAS) in Temuco (Chile), 2012, to 254 DAS in Rauischholzhausen (Germany) 2010. The order of flowering locations for earliest to latest flowering was Temuco (Chile)/Beibei (China)/Gross Gerau/Rauischholzhausen/Giessen (all Germany), whereby the difference was not significant between Giessen and Rauischholzhausen in 2011 (see Fig. 1, Table 1). Although this order was retained during the years 2010–2012, absolute flowering time values of each location also varied significantly between years, reflecting clearly different climatic conditions regarding, temperature and precipitation from 2010 to 2012 and varying day length between the locations (see Fig. 2). Correlations of flowering time between locations show that flowering behaviour was most similar between Gross Gerau and Rauischholzhausen, and most divergent between Giessen and Temuco. This most probably reflects differences in precipitation and day length (Fig. 2). Plant height and seed yield were recorded in all of the German environments, while plant height was also recorded in Temuco. Correlations between flowering time and plant height were always significantly positive (*p* = 0.05) with the exception of Temuco 2012, which was positive, but not significant. Correlations between flowering time and seed yield were negative (with the exception of Giessen 2011) and significantly so for Gross Gerau (all years) and Rauischholzhausen 2012. Correlations between plant height and yield showed no trend and were not significant (correlations see Additional file 1: Table S1).

**Table 1:** Phenotypic means for all locations and years

		Begin of flowering [DAS]								Plant height at flowering [cm]								Seed yield [dt/ha]							
Location	Year	Min	LCL	means	UCL	Max	std	class	lines	Min	LCL	means	UCL	Max	std	class	lines	Min	LCL	means	UCL	Max	std	class	lines
Gross Gerau	2010	218	224.7	225.3	225.9	234	2.9	e	158	60.0	102.4	104.5	106.7	155.0	14.5	g	158	2.2	20.1	21.1	22.1	33.7	5.9	f	158
Rauischholzhausen	2010	235	244.8	245.3	245.9	254	2.13	a	158	100.0	157.3	159.4	161.	5 200.0	16.9	a	158	17.5	47.3	48.3	49.4	81.7	11.3	a	158
Giessen	2011	224	228.3	228.9	229.5	233	2.21	d	158	85.0	111.0	113.1	115.2	138.0	10.8	e	158	3.5	14.8	15.8	16.8	29.4	5.2	g	158
Gross Gerau	2011	221	227.6	228.2	228.7	235	2.38	d	158	95.0	124.7	126.8	128.9	185.0	12.8	c	158	12.8	33.2	34.2	35.2	52.9	8.3	b	158
Rauischholzhausen	2011	210	218.4	219.0	219.6	230	3.88	g	158	105.0	138.5	140.6	142.7	180.0	15.8	b	158	4.8	23.3	24.3	25.3	41.4	6.8	e	158
Temuco	2011	164	171.3	171.9	172.5	202	5.47	i	143	42.3	82.1	84.4	86.6	133.7	16.0	h	144	-	-	-	-	-	-	-	-
Giessen	2012	235	241.1	241.7	242.3	249	2.79	b	156	70.0	102.8	104.9	107.0	142.0	12.5	g	156	0.8	10.9	12.0	13.0	24.2	4.5	h	151
Gross Gerau (rep 1)	2012	227	234.1	234.6	235.2	246	3.77	c	158	82.0	106.5	108.6	110.7	138.0	10.9	f	158	11.1	27.3	28.3	29.3	45.3	7.3	c	156
Gross Gerau (rep 2)	2012	214	221.2	221.7	222.3	231	3.5	f	158	88.0	119.1	121.2	123.4	175.0	13.3	d	158	7.7	24.4	25.5	26.5	44.7	7.3	de	157
Rauischholzhausen	2012	214	221.6	222.2	222.8	236	3.51	f	157	91.0	119.2	121.3	123.5	163.0	12.9	d	158	3.8	25.2	26.2	27.2	46.5	7.6	d	157
Beibei	2012	169	183.8	184.4	184.9	194	4.25	h	151	-	-	-	-	-	-	-	-	-	-	-	-	-	-	-	-
Temuco	2012	157	168.5	169.1	169.7	194	6.51	j	136	61.0	107.4	109.6	111.9	149.7	15.3	f	137	-	-	-	-	-	-	-	-

For every trait, the table gives values for minimum and maximum phenotypic units (Min, Max), lower and upper confidence limits (LCL, UCL), means and standard errors (means, std) as well as the class and the number of lines used to calculate those values

### Population structure

PCA plots using 3 clusters for components 1/2 and 2/3 are shown in Additional file 2: Figure S1. The first four principal components explained 7.04, 6.46, 4.33 and 3.85 % of the genetic variation, respectively. This revealed slight population structure indicated by 3 clusters (see Additional file 2: Figure S1). Identity by descent (IBD) values among clusters 1 and 2 were higher than for the total population, while cluster 3 showed slightly lower IBD values than the total population. This cluster includes three winter-type fodder rapes and one exotic accession and shows greater genetic variance than the population average. *F*_*ST*_ values among cluster pairs revealed highest relatedness between clusters 2 and 3, whereas cluster 1 was approximately equally distant from clusters 2 and 3. This means that cluster 1 clearly separates from clusters 2 and 3, whereas clusters 2 and 3 separate into a defined subpopulation with higher relatedness (cluster 2) and a less defined subpopulation with more diverse material (cluster 3). Total population *F*_*ST*_ was found to be 0.1273, indicating a medium to high relatedness across the overall population. Based on these findings and on further testing of population stratification, GWAS was conducted using a mixed model approach with PC-adjustment.

### Linkage disequilibrium

**Fig. 3:**
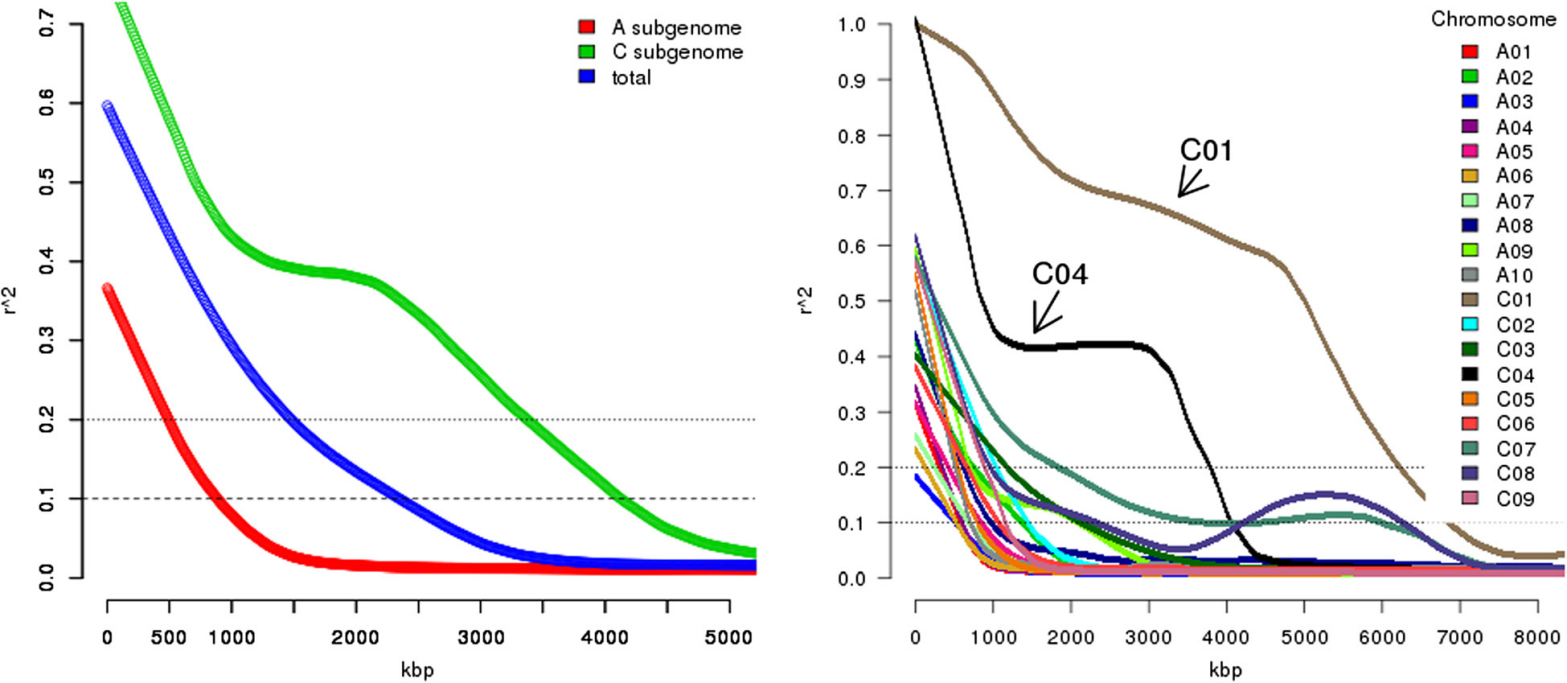
Patterns of linkage disequilibrium (LD) decay. Left: the total genome compared to the respective A and C subgenomes, Right: each of the 19 *B.napus* chromosome separately. Chromosomes C01 and C04 are indicated by arrows

Mean pairwise *r*^*2*^ for all chromosomes was found to be 0.105. When comparing the decay of LD among the two subgenomes, it is evident that the C subgenome shows a much slower average LD decay than the A subgenome (Fig. 3). Whereas LD in the A subgenome drops to less than 0.2 within 494.8 kbp, in the C subgenome LD does not drop under 0.2 until an average of 3389.5 kbp. When investigating into this phenomenon by chromosome, it becomes evident that chromosomes C01 and C04 are the major causes for the slow LD decay in the C genome, with additional contributions from chromosomes C07 and C08 (Fig. 3). This means that chromosomes C02, C03, C05 and C09 show a behavior similar to the A genome. LD decay on chromosome C08 shows an unexpected shape with a clearly visible peak centered at 5256.0 kbp, which might represent a signature of co-selection. On chromosome C07, there is a similar, but less pronounced peak centered on 5414.1 kbp.

### Association analysis

#### Onset of flowering

**Fig. 4:**
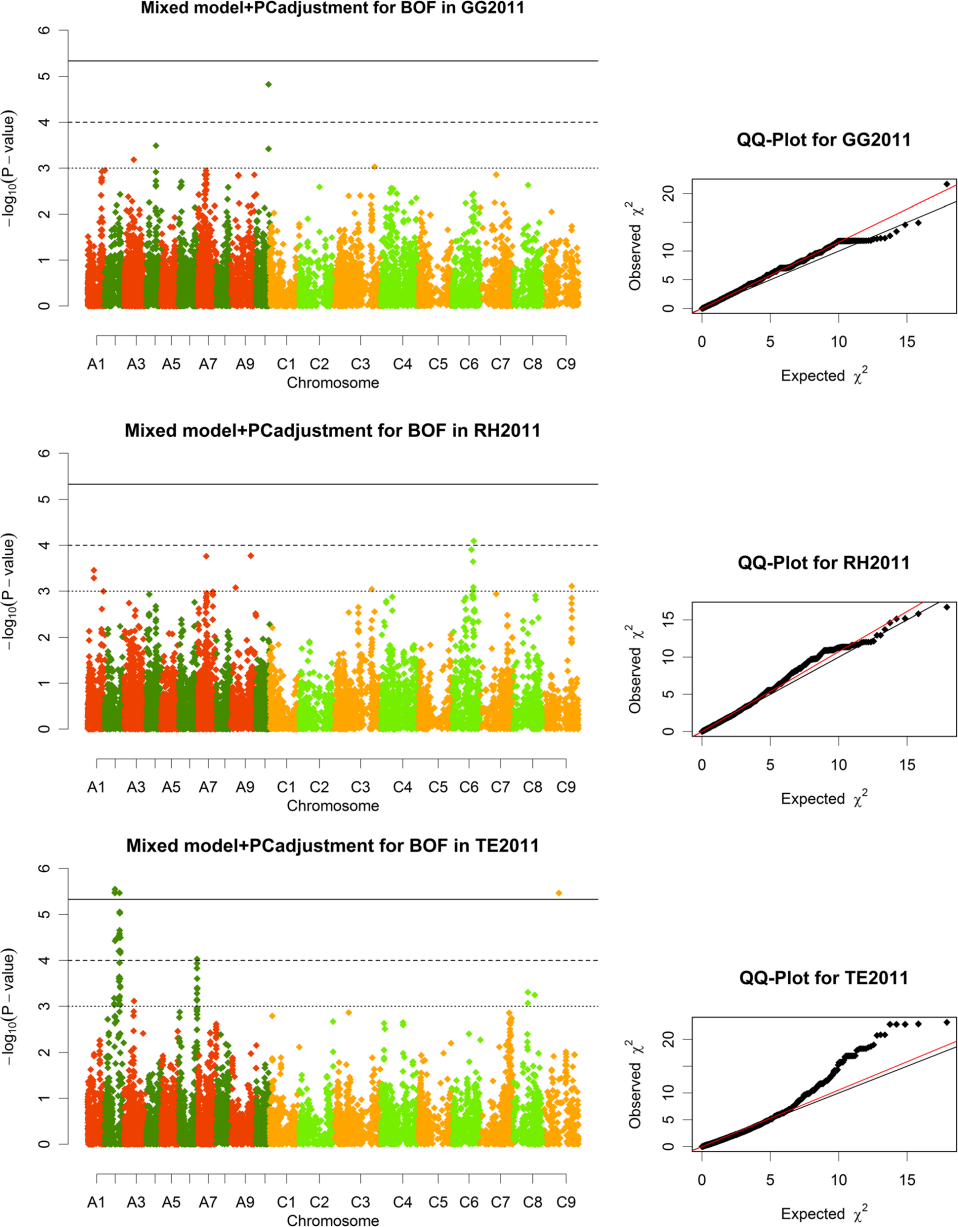
Manhattan plots and QQ plots. Describing marker-trait associations for absolute values of the onset of flowering (BOF-ABS) in the three divergent environments Gross Gerau, Rauischholzhausen (both Germany) and Temuco (Chile) in 2011. Associations were calculated using a mixed model with PC adjustment, using 21,623 single-copy, genome-wide SNP markers. The solid line represents the significance threshold (*p* = 0.1) for associations after Bonferroni correction, the dashed line represents the level considered as significant in this analysis (−log_10_(*p*) > 4), and the dotted line represents the threshold for suggestive associations(−log_10_(*p*) > 3). The A and C subgenomes are displayed in a slightly different color code (dark: A subgenome, bright: C subgenome)

Association analysis for beginning of flowering (BOF) in the context of absolute flowering time (BOF-ABS) and relative flowering time (BOF-REL, see Methods for definition) defined 62 and 47 regions of interest, respectively, 8 of which overlapped. Examples of Manhattan plots for BOF-ABS are shown in Fig. 4, along with their quantile-quantile (QQ) plots. All regions were compared to the positions of known *B. napus* gene models which were published in [20]. Gene ontology terms were queried for flowering-related terms like “flower development”, “vegetative to reproductive transition”, “photoperiod” or “vernalisation” (see methods for full list). For BOF-ABS a total of 181 flowering related genes were identified in 49 candidate regions, while 13 regions were left unexplained by flowering related gene ontology. For BOF-REL, 232 flowering related genes were identified in 38 regions of interest, leaving 9 regions unexplained. A total of 39 genes overlapped between both analyses. A full list of all candidate genes with their closest and highest associating SNP markers, including their phenotypic effects, is shown in Additional file 3: Table S2. For BOF-ABS, the list contains 31 transcription factors, 2 translation initiation factors, 1 translation elongation factor and 18 protein kinases. Furthermore, 2 histones and 11 histone interacting factors were among the candidates. For BOF-REL we found 42 transcription factors, 3 translation initiation factors and 19 protein kinases, as well as 25 histone interacting factors.

Among the detected transcription factors within BOF-ABS candidate regions, orthologs to 7 *A.thaliana* agamous-like (AGL) transcription factors were found, 6 of which are known to play a role in determining floral meristem identity [31]. In the analysis of BOF-REL we detected 8 orthologs to agamous-like transcription factors as well as a copy of the transcriptional co-repressor *LEUNIG (BnaC01g05930D)* which is known to negatively regulate *AGAMOUS (BnaC03g62970D,* found for BOF-ABS*)*. Copies of *Bna.CO* and *Bna.FLC* were not detected among the candidate regions, although an ortholog of *FRIGIDA* (*BnaA03g13320D*), a central vernalisation regulator, was detected in the Chinese environment in BOF-REL, in relative proximity to a copy of *FLC*. A summary of interesting candidate genes, including the abovementioned, is given in Table 2. Overall, we detected only rare examples of early-acting flowering regulators, while late-acting flowering regulators and floral homeotic genes were more predominant among the associated regions.

**Table 2:** Examples of candidate genes within LD blocks associated with absolute flowering time (BOF-ABS) and relative flowering time (BOF-REL)

Trait	Chromosome	Marker at association peak	-loglO(p)	Candidate gene	Gene description	distance [kbp]
BOF-ABS	chrA02	Bn-A05-p9078281	5.55	BnaA02g20680D	luminidependens	10.79
BOF-REL		Bn-A02-p20678898	5.27			138.89
BOF-ABS		Bn-A05-p9078281	5.55	BnaA02g22450D	SET DOMAIN PROTEIN 18	413.00
BOF-ABS		Bn-A05-p9078281	5.55	BnaA02g27250D	SEPALLATA 4	68.37
BOF-REL		Bn-A02-p20678898	5.27			68.37
BOF-REL	chrA03	Bn-A03-p6710651	4.46	BnaA03gl3320D	frigida	0.63
BOF-ABS		Bn-A03-p21357849	3.13	BnaA03g40220D	protein agamous like 71	28.50
BOF-ABS	chrAO8	Bn-A08-p7018554	3.24	BnaA08g06080D	NUCLEAR FACTORY SUBUNIT A8	23.74
BOF-ABS	chrA09	Bn-A01-p9004629	5.82	BnaA09g05410D	AGAMOUS LIKE 32	61.46
BOF-ABS		Bn-A01-p9004629	5.82	BnaA09g05500D	FRUITFULL	41.26
BOF-ABS		Bn-A01-p9004629	5.82	BnaA09g05570D	glycine rich rna binding protein 3	66.08
BOF-ABS		Bn-A01-p9004629	5.82	BnaA09g05900D	FOREVER YOUNG FLOWER	199.43
BOF-ABS		Bn-A09-p33006858	3.03	BnaA09g44920D	NUCLEAR FACTOR Y SUBUNIT AS	9.89
BOF-ABS	chrA10	Bn-A10-p9065520	3.51	BnaA10gl2840D	HISTONE H2A 6	3.08
BOF-ABS		Bn-A10-p9065520	3.51	BnaA10gl2870D	HI STONE HTB4	4.47
BOF-REL	chrC0l	Bn-scaff_15838_5-p329539	4.25	BnaC01g05930D	transcriptional corepressor leunig	4.65
BOF-REL		Bn-scaff_16874_2-p29251	4.01	BnaC01gl7380D	gibberellin 20-oxidase	372.20
BOF-REL		Bn-scaff_16874_2-p29251	4.01	BnaC01gl7400D	protein agamous-like 71	350.53
BOF-REL		Bn-scaff_16874_2-p29251	4.01	BnaC01gl7410D	FLOWERING WAGENINGEN	272.47
BOF-REL	chrC02	Bn-A05-p8798628	3.69	BnaC02g27000D	luminidependens	1006.78
BOF-REL	chrC03	Bn-scaff_28774_l-p25229	3.51	BnaC03gl5810D	protein agamous-like 71	3.14
BOF-REL		Bn-scaff_28774_l-p25229	3.51	BnaC03gl5820D	protein agamous-like 71	10.97
BOF-REL		Bn-scaff_28774_l-p25229	3.51	BnaC03gl5830D	protein agamous-like 42	21.08
BOF-ABS		Bn-scaff 17044 l-p526219	3.41	BnaC03g48540D	BTB AND TAZ DOMAIN PROTEIN 4	9.39
BOF-ABS		Bn-scaff 16182 l-p296671	3.56	BnaC03g62970D	AGAMOUS	287.72
BOF-ABS		Bn-scaff 16182 l-p296671	3.56	BnaC03g64560D	terminal flowering 1 protein 1	143.12
BOF-ABS	chrC05	Bn-scaff 17869 l-p926723	3.37	BnaC05g21200D	SEPALLATA3	26.50
BOF-REL	chrC06	Bn-scaff _17799_l-p2161244	3.76	BnaC06g35180D	glycine-rich rna-binding protein 5	52.75
BOF-REL	chrC08	Bn-scaff _16361_l-pl466598	3.45	BnaC08g28320D	agamous-like mads-box protein agl18	10.94
BOF-REL		Bn-scaff _16361_l-p2115007	5.58	BnaC08g29520D	shatterproof1	41.99
BOF-REL		Bn-scaff _16361_l-p2115007	5.58	BnaC08g29530D	shatterproof1	52.09
BOF-REL	chrC09	Bn-scaff _17799_l-p3049743	3.57	BnaC09g34850D	sensitivity to red light reduced protein	848.30
BOF-REL		Bn-scaff _17799_l-p3049743	3.57	BnaC09g35410D	pseudo-response regulators	375.12
BOF-REL		Bn-scaff _17799_l-p3049743	3.57	BnaC09g35430D	ap2-like ethylene-responsive transcription factor toe2	357.37

Genes with gene ontology (GO) terms related to flowering were regarded as candidates if they shared an LD block (*r* ^*2*^ > 0.5) with one or more trait-associated markers, For each gene the most strongly trait-associated neighbouring marker(s) are named along with the gene description

### Plant height

**Fig. 5:**
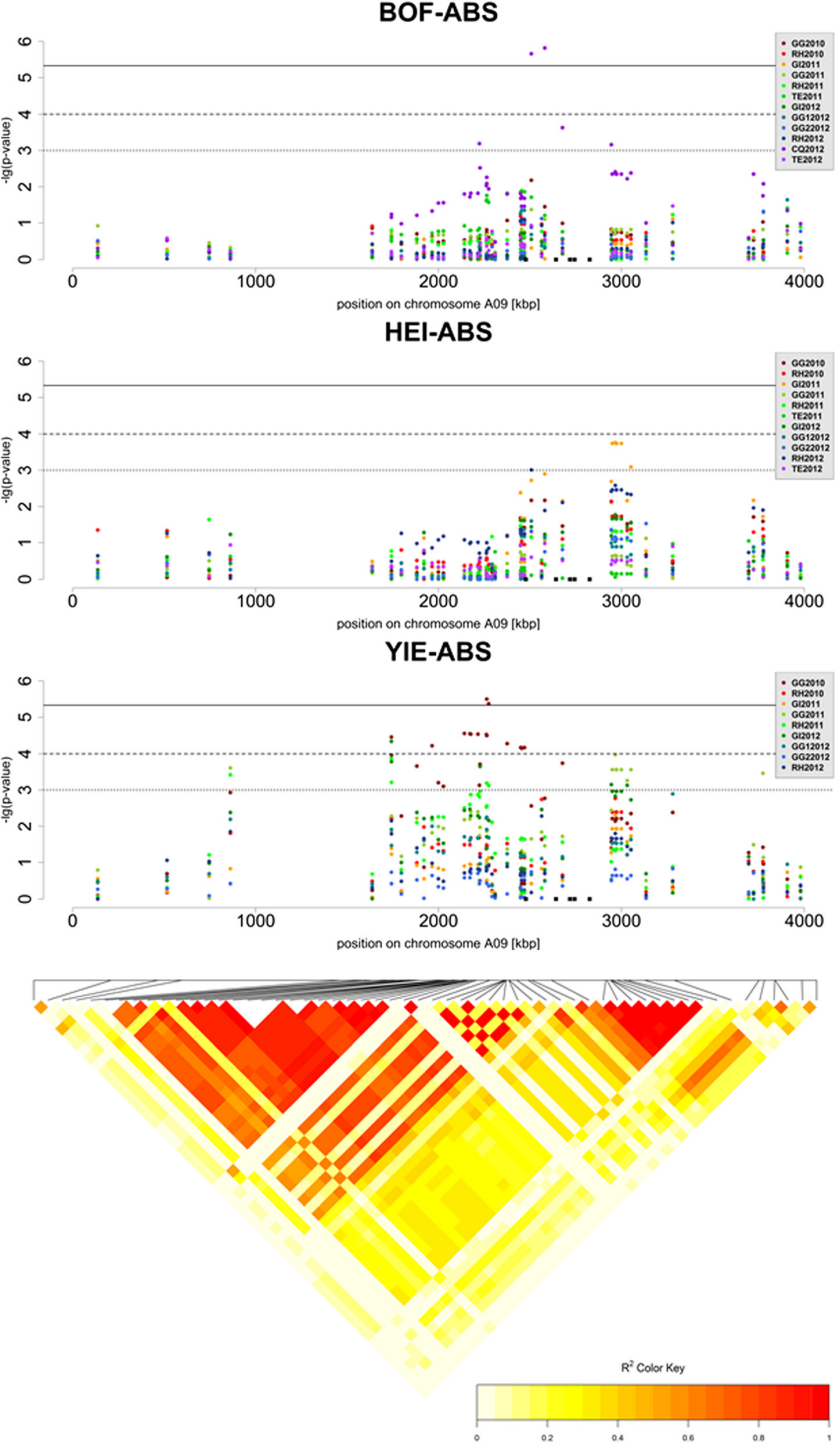
Local representation of marker-trait associations with absolute values for flowering time (BOF-ABS), plant height (HEI-ABS) and seed yield (YIE-ABS) in a region of *B.napus* chromosome A09. Each environment is represented in a unique colour consistent between representations, as shown by the colour key. The solid line represents the significance threshold 0.1 after Bonferroni correction, the dashed line represents the significance threshold in this analysis (−log10(*p*) > 4), and the dotted line represents the threshold for suggestive associations (−log10(*p*) > 3). The positions of 5 candidate genes within the peak interval are indicated by black squared dots. The representation below shows the local conservation of linkage disequilibrium (*r* ^*2*^) among the SNPs in the trait-associated region is represented by a heat map

For plant height (HEI-ABS), 69 regions of interest were found to be associated with the available phenotype data from the environments in Chile and Germany. Gene ontology terms were searched for terms like “photosynthesis”, “catabolic”, “cell growth” or “development”, resulting in a list of 712 genes covering 65 from 69 regions of interest (see methods for full list of GO terms). Out of the regions of interest for plant height, 17 overlapped with regions associated with BOF-ABS, containing for example copies of the agamous-like transcription factors *AGL32 (BnaA09g05410D), FRUITFUL (FUL; BnaA09g05500D, BnaC02g41870D)* and *FOREVER YOUNG FLOWER (FYF; BnaA09g05900D)* (Fig. 5). Overall, we detected 8 AGL transcription factors, including a copy of *SEPALLATA 3 (SEP3; BnaA09g28040D)* differing from the copy found for BOF-ABS *(BnaC05g21200D)*. The candidate regions also contained orthologs of the flowering genes *GIGANTEA* (*GI; BnaA09g30390D*), *TEMPRANILLO1* (*TEM1; BnaA07g35130D*) and *FPA (BnaC08g29740D)*. Moreover, we found orthologs to the squamosa-promotor binding-like proteins *SPL1 (BnaA05g00780D), SPL4 (BnaC06g10070D), SPL10 (BnaA09g27950D)* and *SPL11(BnaA09g27960D)*. To summarize, a high overlap of associating regions was observed between flowering time and plant height. Moreover, some regions associated with plant height contained flowering-related genes for which associations to flowering time itself were not detected.

### Yield

For yield (YIE-ABS), 36 regions of interest were defined by the associations detected with the available phenotype data from the German environments (no data available for Chile and China). Of those, 6 regions overlapped with BOF-ABS and 4 with HEI-ABS, while 1 region on chromosome A09 overlapped between all of them (Fig. 5). Gene ontology terms corresponding to the genes among these regions were searched for terms like “lipid”, “terpenoid”, “biosynthetic process” or “embryo (sac) development” (see methods for full list of GO terms). The 568 detected genes covered 34 from 36 regions of interest. Noteably, 31 % of the candidate genes within these regions had an orthologous copy in another candidate region, whereas for flowering and plant height this was the case for only 8 % (BOF-ABS), 9 % (BOF-REL) and 9 % (HEI-ABS) of the detected genes. This was mainly due to several regions of interest being homoeologous, for example on chromosomes A03/A09/C09, A04/C04 and A05/C05, but also due to adjacent duplicated genes within the same region. Homoeology between associated regions was not observed for BOF-ABS, and only a very short segment on chromosomes A02/C02 was found to be homoeologous between regions associated with BOF-REL (containing the two copies of *LUMINIDEPENDENS; BnaA02g20680D* and *BnaC02g27000D*). HEI-ABS showed 3 regions homoeologous to each other on chromosomes A03/C03, A06/C05 and A09/C02, respectively. Examples of duplicate copies of a gene or gene family are given in Table 3.

**Table 3:** Examples of duplicated candidate genes within LD blocks associated with yield

Chromosome	Marker at association peak	-loglO(p)	Candidate gene	Gene description	distance [kbp]
chrA03	Bn-A03-p21075664	3.44	BnaA03g39700D	methylthioalkylmalate synthase	26.91
	Bn-A03-p21075664	3.44	BnaA03g39710D	methylthioalkylmalate synthase	24.05
	Bn -A03-p21075664	3.44	BnaA03g39720D	methylthioalkylmalate synthase	18.82
	Bn-A03-p21075664	3.44	BnaA03g39790D	methylthioalkylmalate synthase	4.24
	Bn-A03-p21075664	3.44	BnaA03g39860D	protein cobra-like	74.66
chrA04	Bn-A04-p9991606	4.05	BnaA04gl3120D	cytochrome p450	12.10
	Bn-A04-p9991606	4.05	BnaA04gl3160D	cytochrome p450 71b2	8.10
chrA09	Bn-A09-pl670189	5.50	BnaA09g02980D	glutamate receptor	640.82
	Bn-A09-pl670189	5.50	BnaA09g02990D	glutamate receptor	656.01
	Bn-A09-pl670189	5.50	BnaA09g03000D	glutamate receptor	659.35
	Bn-A09-pl670189	5.50	BnaA09g03020D	glutamate receptor	663.14
	Bn-A09-pl670189	5.50	BnaA09g03560D	cysteine synthase d2	73.83
	Bn-A09-pl670189	5.50	BnaA09g03580D	cysteine synthase d2	70.07
	Bn-A09-p3047090	3.97	BnaA09g05570D	protein cobra-like	213.15
chrC02	Bn-scaff_17109_l-p683400	3.68	BnaC02g38580D	glutamate receptor	110.57
	Bn-scaff_17109_l-p683400	3.68	BnaC02g38590D	glutamate receptor	107.50
chrC04	Bn-scaff_15798_l-p725659	4.25	BnaC04g36000D	gdsl esterase lipase at2g23540-like	3.17
chrC07	Bn-scaff_17326_l-p536514	4.23	BnaC07gl0060D	cytochrome p450	310.08
	Bn-scaff_17326_l-p536514	4.23	BnaC07gl0090D	cytochrome p450	315.95
	Bn-scaff_17326_l-p536514	4.23	BnaC07gl0250D	gdsl esterase lipase	611.35
	Bn-scaff_17326_l-p536514	4.23	BnaC07gl0260D	gdsl esterase lipase	614.33
	Bn-scaff_17326_l-p536514	4.23	BnaC07gl0320D	gdsl esterase lipase	617.31
	Bn-scaff_16069_l-p3940746	3.77	BnaC07g39560D	protein trichome birefringence-like 18	0.79
chrC08	Bn-scaff_16069_l-p3940746	3.77	BnaC08g20710D	protein trichome birefringence-like 18	1.13
chrC09	Bn-scaff_19783_l-p379086	5.36	BnaC09g02400D	glutamate receptor	1501.80
	Bn-scaff_19783_l-p379086	5.36	BnaC09g02410D	glutamate receptor	1481.75
	Bn-scaff_19783_l-p379086	5.36	BnaC09g02430D	glutamate receptor	1476.34
	Bn-scaff_19783_l-p379086	5.36	BnaC09g02940D	cysteine synthase d2	1164.69
	Bn-scaff_19783_l-p379086	5.36	BnaC09g02970D	cysteine synthase d2	1160.62
	Bn-scaff_19783_l-p379086	5.36	BnaC09g05090D	protein cobra-like	148.92
	Bn-scaff_19783_l-p379086	5.36	BnaC09g05120D	protein cobra-like	156.02

Genes with gene ontology (GO) terms related to flowering were regarded as candidates if they shared an LD block (*r* ^*2*^ > 0.5) with one or more trait-associated markers, For each gene the most strongly trait-associated neighbouring marker(s) are named along with the gene description

Some classical flowering genes were also found among those regions, for example two copies of *FUL (BnaA03g39830D, BnaA09g05510D)* and *FLOWERING PROMOTING FACTOR 1 (FPF1; BnaA09g04780D, BnaC09g04250D)*, and one copy each of *TFL1 (BnaA10g26300D)* and *FT (BnaC04g14850D)* along with two copies of *PSEUDO RESPONSE REGULATOR 5* (*PRR5; BnaA09g04840D, BnaC09g04380D*) and one copy of *PRR1 (BnaC09g05250D)*.

Overall, homoeologous chromosome regions were commonly associated to yield, and many of these regions carry copies of flowering-time candidate genes. This indicates a parallel selection among *B. napus* subgenomes.

### Multi-trait associations

For multi-trait comparative analysis (COMP) we defined 68 regions of interest, taking into account the relative phenotypic values only for German environments in which all traits were measured. The regions of interest showed at least 1 suggestive association for one trait and at least 1 supporting association for another trait. We considered genes with GO terms referring to all traits under study as described before. This identified 739 candidate genes covering 67 from 68 regions of interest. Among the candidates were orthologs to well-known components of the circadian clock like *CIRCADIAN CLOCK ASSISTED 1* (*CCA1; BnaA05g01050D*) and *TIME FOR COFFEE* (*TIC; BnaC03g42780D*), which both occurred in a region inversely affecting plant height and yield in Gross Gerau in 2011. We moreover detected a copy of *FT* that was inversely associated with plant height and yield in Rauischholzhausen in 2011 (*BnaA07g25310D*). The 3 AGL transcription factors *Bna.AGL6, Bna.AGL8/FUL, Bna.AGL65* were again detected. *AGL6* is a regulator of *FLC* and *FT* [32], while *AGL65* is necessary for pollen development [33]. *AGL6* and *FUL* (the same copy as in YIE-ABS) are found in regions affecting all traits.

Copies of the he three SPL orthologs *SPL1*, *SPL4* and *SPL8 (BnaA10g00110D)* were also detected in COMP. For *SPL1* and *SPL4* the same copies were detected as for HEI-ABS, while *SPL8* is known to regulate anther development. Moreover, we detected a copy of *LIGHT-DEPENDENT SHORT HYPOCOTYLS* (*LSH1; BnaC05g47190D*), a phytochrome-dependent regulator of seedling development, in a region also associated with contrasting effects on flowering and height in Giessen in 2011 (early/small; late/high).

### Central flowering regulators

Except for a homolog of *FRI* on chromosome A03 *(BnaA03g13320D)*, none of the central flowering regulators (e.g., *Bna.FT*, *Bna.CO* or *Bna.GI*) or central vernalisation genes (e.g., *Bna.FLC*) were found among the candidate genes for the onset of flowering (BOF-ABS/ BOF-REL). Moreover, the A03 *FRI* copy was only detected in association with BOF-REL in the sub-tropical environment of Beibei 2012. A paralog of *FLC* was found in a region with suggestive association, also in Beibei 2012. When checking for closest marker associations of further copies of *FLC*, some copies (on chromosomes *A01*, *A02* and *C02*) showed supporting, but no suggestive or significant associations in either BOF-REL or BOF-ABS. On the other hand, we found homologs of *FUL (BnaA09g05500D, BnaC02g41870D)*, *GI* (*BnaA09g30390D*) and *TEM1* (*BnaA07g35130D*) for HEI-ABS, *TFL1 (BnaA10g26300D), FT* (*BnaC04g14850D*) for YIE-ABS, and *FT* (*BnaA07g25310D*) for COMP.

**Fig. 6:**
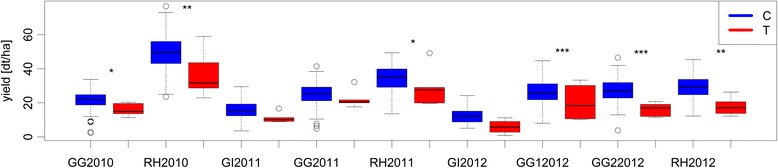
Boxplots showing yield values for genotypes carrying the FT-C-allele (blue) and the FT-T-allele (red) for each location and year. The environments are abbreviated as follows: Giessen (GI), Gross Gerau (GG), Rauischholzhausen (RH) with their respective year of harvest. Significant differences tested with the student’s *t*-test are labelled (*p < 0.05, **p < 0.01, ***p > 0.001)

Resequencing all copies of *B. napus FT* for a subset of 121 genotypes using a sequence capture approach (methods according to [34]) revealed non-synonymous SNP variation for *BnaA07g25310D* (R21Q, I49L) and *BnaC04g14850D* (H81Y). Whereas *BnaA07g25310D* exhibited heterozygous SNP calls, suggesting more than one homologous locus, clear genotyping calls were obtained for *BnaC04g14850D*. The T allele was associated with later flowering; taller plants and inferior yield compared to the C allele, with significant effects in different environments for flowering, plant height and yield (see Fig. 6).

Resequencing across all copies of *Bna.CO* revealed 10 nonsynonymous SNPs for *BnaA03g27860D* (4), *BnaA10g18430D* (1), *BnaC03g32910D* (1) and *BnaAnng39160D* (4). Testing those SNPs for association to flowering time, plant height and yield revealed associations for SNPs on *BnaA10g18430D* (E203D, associated with flowering time and yield), *BnaC03g32910D* (E79Q, associated with flowering time) and *BnaAnng39160D* (H165Q, associated with plant height, Q210H, associated with flowering time and yield). For *Bna.SOC1*, 4 nonsynonymous SNPs were detected for *BnaC04g53290D* (1), *BnaC05g00840D* (1) and *BnaCnng45490D* (2). Testing for associations revealed associations for SNPs on *BnaC05g00840D* (E191D, associated with plant height) and *BnaCnng45490D* (D122E, associated with all traits). We detected supporting associations only for SNPs closest to *BnaA03g27860D* (COMP, yield) and *BnaC03g32910D* (COMP, height), whereas no associations were detected for SNPs closest to *BnaA10g18430D*, *BnaC09g46500D*, *BnaA04g26320D* and *BnaC05g00840D* . LD between the adjacent flanking SNPs was low to very low (*r*^*2*^ = 0.17 for *BnaA03g27860D*, 0.002 for *BnaA10g18430D*, 0.42 for *BnaC03g32910D*, 0.002 for *BnaC09g46500D*, and *r*^*2*^ = 0.295 for *BnaC04g53290D*).

### Allelic effects

**Fig. 7:**
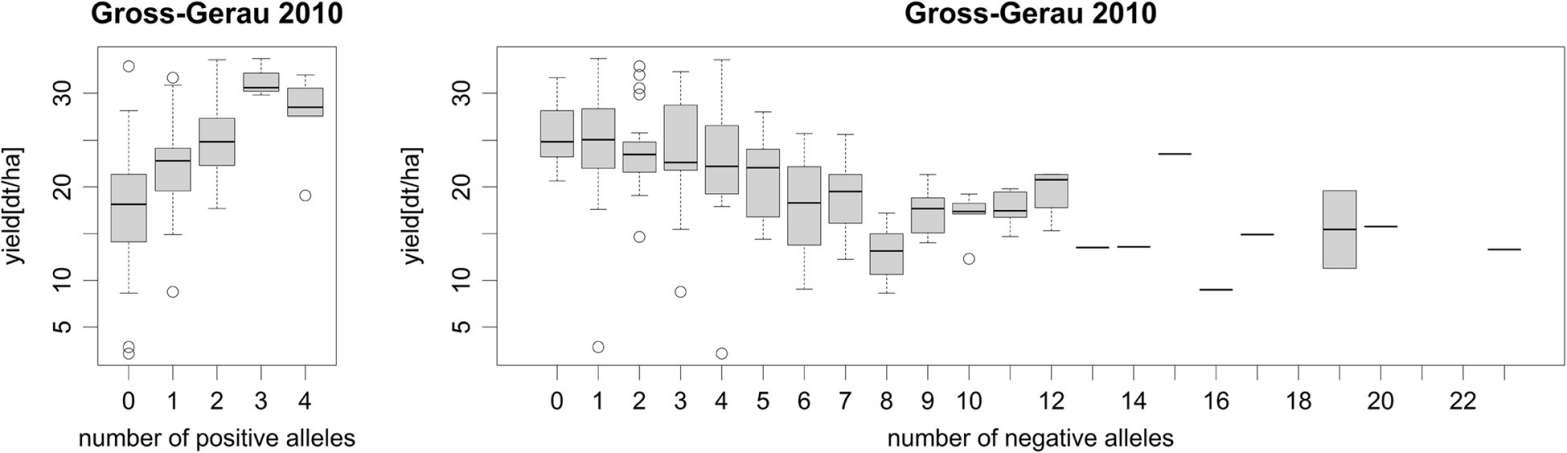
Boxplots showing yield values for genotypes carrying the specified number of positive and negative alleles for Gross-Gerau in 2010

Testing for allelic effects in BOF-ABS, HEI ABS and YIE-ABS, we observed significant additive effects for plant height and yield but not for flowering time. Whereas the phenotypic effect was subtle for plant height, it was strong for yield. Generally, the major allele induced an early, short, high-yielding phenotype, whereas the number of major alleles leading to late, high, low-yielding plants was small. This suggests that selection within this population has strongly driven phenotype towards early-flowering, smaller and better yielding plants (see Fig. 7, Additional file 4 Figure S2 for an example).

### Gene ontology enrichment

**Fig. 8:**
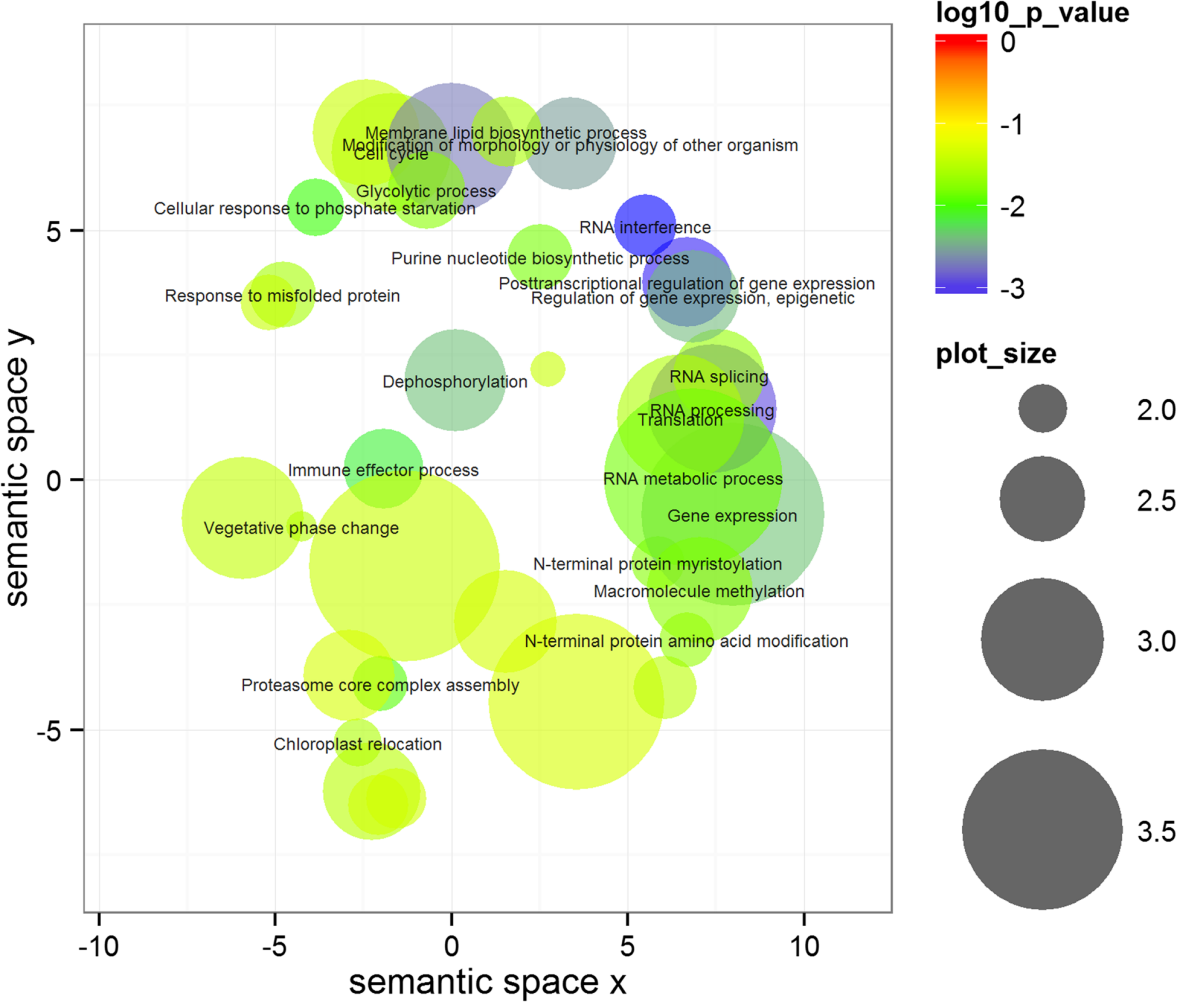
Gene ontology scatterplot constructed with REVIGO in R [39] for all genes in the associated regions for absolute values of flowering time (BOF-ABS). The scatterplot accounts for all GO terms with a count of min. 10., and were constructed using the Arabidopsis thaliana GO term database, using a similarity of 0.5, with SimRel as semantic similarity measure. Colours indicate the p-value of enrichment according to the legend. The size of each bubble reflects the count of each term among the enriched term list. Terms with a –log(*p*) > 1.5 are ascribed to their bubbles

GO enrichment analysis for all 1733 genes among the detected regions in BOF-ABS revealed significant enrichment of terms referring to post-transcriptional regulation of gene expression, for example RNA interference, miRNA regulation and RNA methylation. Terms related to epigenetic regulation were also significantly enriched, like methylation-dependent chromatin silencing, as well as dephosphorylation processes, proteasome core complex assembly and cell cycle (see Fig. 8). Vegetative phase change and response to stimulus were also among the significantly enriched GO terms. For BOF-REL, the most enriched terms among the 3133 genes were genes connected with response to oomycetes and negative regulation of programmed cell death. Petal development and aging were also enriched (Additional file 5: Figure S3).

GO enrichment analysis carried out for 2249 genes among regions of interest for HEI-ABS revealed the importance of small molecule metabolic processes for plant height, including monocarboxylic acid metabolism, cellular ketone metabolic process, carbohydrate metabolic process, alcohol catabolic process and the electron transport chain. The second group of enriched GO terms included processes involved in developmental regulation, like lateral root development, cell fate commitment and leaf senescence. We found also terms referring to virus and insect responses, to regulation of protein metabolic processes, as well as the cellular biogenic amine metabolism (Additional file 5: Figure S3).

The GO enrichment performed for the 2144 genes among the interesting regions for YIE-ABS showed a strong enrichment for metabolic pathways like triterpenoid metabolism, steroid metabolism or regulation of lipid metabolic processes. Pathways like sulfur, phosphorus and nitrogen compound metabolism were also enriched, as well as positive regulation of developmental processes, cell proliferation and gene expression (Additional file 5: Figure S3).

For 2162 genes detected in COMP, we found a strong enrichment for response to environmental factors like abiotic, biotic or endogenous stimuli, hyperosmotic response or response to heat. Moreover there was enrichment for biosynthesis of both small and large molecules, (e.g., flavonoids), immune system processes and macromolecule methylation (Additional file 5: Figure S3).

## Discussion

The diversity set used to assemble our winter-type *B. napus* panel was designed to represent the available breeding gene pool in the species [35, 36]. Genotyping the selected 158 winter lines with the Brassica 60 k Illumina® Infinium SNP array allows deep insight into the genetic variation present in this subset, although a large part of genotyping information is lost due to ambiguity in the reference genome. Chequerboard patterns in LD (Fig. 5) also indicate that there still may be issues concerning the reference assembly, as markers linked to each other are separated by ones which are not linked to them. Another explanation for this phenomenon could be rearrangements in the genomes of some lines, a common phenomenon in *B. napus* [20], which could decrease local LD. Since almost all accessions were adapted winter-type materials, the relatedness within the selected population was medium to high, reflecting the low genetic diversity expected within this gene pool [37]. The narrow genetic diversity was reflected by the relatively low associations detected for all traits. The low effects of associated loci also reflect the nature of the traits under investigation, which are influenced by a large number of different small-effect loci, reducing single contributions. The investigation of allelic effects for absolute values of flowering time, plant height and yield revealed that the population was subjected to strong selection towards an early flowering, small growing and high yielding phenotype. The results suggest that there is still potential to increase yield by pyramiding positive alleles. This might also be possible with regard to plant height, but does not hold true for flowering time. Flowering time genes do not act in parallel, but rather in a complex adaptive network, therefore effects are not stable over different environments. The suggestion that pyramiding of haplotypes may have a positive effect on yield is mirrored by observations of large homoeologous regions associated to yield (see below), which also points to parallel selection. All the same, the number of positive alleles is limited, demonstrating the importance of increasing diversity to ensure stable yield progress in changing environments [38].

Due to the low association levels, we used a significance cutoff based on FDR, to avoid loss of interesting loci, and used different approaches for detection of important regulators. Calculating genome-wide associations with absolute values for the onset of flowering, plant height and yield allowed us to find regions responsible for differences between genotypes (BOF-ABS, HEI-ABS, YIE-ABS), whereas calculating with the difference of the respective absolute value from the genotypic mean allowed us to detect regions responsible for differential population behaviour in different environments (BOF-REL). Comparisons between such relative association analyses for the same set of environments allowed detection of loci with adaptive potential (COMP).

**Fig. 9:**
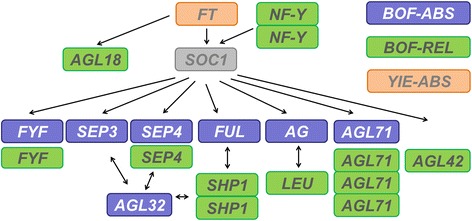
Schematic representation of interactions in the Arabidopsis *SOC1* regulation module for all respective candidate genes. The scheme includes all interaction partners which were detected as candidate genes for the onset of flowering in this study. For clarity, the *FT* copy detected in YIE-ABS was also added. Simple arrows represent regulation, double arrows represent interaction

The GO term enrichment analyses enabled us to identify the most important biological processes influencing the traits under study. Although the function of candidate genes cannot be confirmed without further reverse genetic analyses, this method allows dissection of trait regulation into pathways even without knowing the exact genetic players [39]. For BOF-ABS, the GO enrichment analysis revealed a superordinate influence of post-transcriptional gene expression regulation on floral initiation. This underlines the particular importance of epigenetic processes like methylation and RNA interference in determination of flowering time. Epigenetic factors play a huge role in adaptive traits, as for example response to abiotic or biotic stress [40]. This is consistent with our results, as non-optimal conditions like pests, water/nutrient deficiency or temperature stress are expected to play a significantly higher role in field trials than in the controlled conditions under which experiments for gene function are performed. This may in part explain why our analysis of flowering time detected only a few classical flowering time genes that do not explain all detected associations. Another reason may lie in the population, which might have been strongly selected for certain alleles. All the same, detection of some of those genes gives hints to pathways that play a role in flowering control under field variable conditions. The *A. thaliana* homologs of the detected candidates *Bna SEP3, Bna SEP4, Bna FUL, Bna.AGL42, Bna.AGL71* and *Bna AG* are all regulated directly or indirectly by *SOC1* [41, 42], which in turn is regulated by *NUCLEAR FACTOR Y.* We therefore propose that the *SOC1* regulatory pathway has a major impact on flowering time determination of winter-type *B.napus* under field conditions (Fig. 9). Sequencing for all *Bna.SOC1* copies in a subset of 121 genotypes revealed a low functional diversity. This indicates *Bna.SOC1* may be highly conserved due to its central role. The only localized *Bna.SOC1* copy that shows functional variation (*BnaC04g53290D*) lay in a region which was not associated to any trait at all. Two copies which could not be assigned to a specific location due to the ambiguity in the reference assembly carried functional variation which was associated to the phenotypic variation.

Moreover, a number of candidate genes detected in BOF-REL, like *Bna.FWA, Bna.TOE2* and *Bna.GRP3* [43, 44], may relate to epigenetic control of flowering. Copies of *TFL1* have recently been found to delay flowering time and increase seed yield in *B.napus* [45].

The GO enrichment for BOF-REL revealed a prevalence of developmental GO terms like negative regulation of programmed cell death, petal or xylem development and aging, indicating that variation at flowering loci impacts many genes downstream of the known regulatory cascades. References to small RNA regulation coincide with the detection of similar AGL transcription factors from the *SOC1* regulation module (*Bna.SEP4, Bna.AGL42, Bna.AGL71, Bna.LEUNIG*) to those identified for BOF-ABS. Interestingly, we also detected two adjacent copies of *Bna.AGL1/SHP1*, whose *A. thaliana* homolog was found to be an antagonist of *FUL* in fruit dehiscence [46], indicating that that this gene might also interact with *FUL* in flowering (Fig. 9).

The only basic flowering time gene associated with BOF-REL was *Bna.FRI*, a well-described vernalisation regulator, in the Chinese environment. *Bna.FRI.a*, corresponding to *BnaA03g13320*, has already been established as a major flowering time determinator in *B.napus* [47]. *Ath.FRI* was also found before to play a role in climatic adaptation [1, 48]. As vernalisation is a quantitative process [49], we hypothesize that, within gene pools adapted to cold winter conditions, functional variation in central vernalisation genes may only play a role when temperatures are barely inductive for vernalisation, but not in environments with long periods of temperatures around and below freezing. Natural variation in vernalisation requirement was previously observed in Arabidopsis [50]. Similarly, we were also unable to detect significant associations with central integrators of the photoperiod pathway like *CO* or *GI*.

GO enrichment analysis for HEI-ABS and YIE-ABS revealed the primary importance of metabolic efficiency for plant height and yield. Whereas small molecule catabolism and the electron transport chain were implicated in control of plant height, the metabolism of higher-order molecules like triterpenoids or lipids appeared to be more influential in regulation of seed yield. Both traits enriched GO terms related to nitrogen metabolism, whereas phosphorus and sulfur metabolism were only enriched for yield, putatively in the context of lipid and glucosinolate metabolism. This may reflect the generally more advanced breeding level and higher yields of modern oilseed rape forms which also have high oil and low seed glucosinolate contents.

Although HEI-ABS and YIE-ABS give a similar GO enrichment, the overlap of associated regions was the lowest between all traits, while the highest overlap was observed for BOF-ABS and HEI-ABS. This is consistent with the respective phenotypic correlations, where we could not see a correlation trend between plant height and yield, but a significantly positive correlation between the onset of flowering and plant height was observed. This indicates that the switch to the reproductive phase determined plant height, however plant height was neither decisive nor indicative for seed yield. All the same, there was a negative correlation between the onset of flowering and yield, supporting the assumption that flowering time influences both traits independently of each other. Notably, some of the overlapping regions contained genes from the *SOC1* regulation module (*Bna.FUL, Bna.FYF*).

It was striking that for YIE-ABS, more than 30 % of the candidates were detected with more than one copy, caused by several regions of interest being homoeologous, indicating co-expression of those genes between the subgenomes. The highest and largest regions of interest were found on homologous regions of chromosomes A09 and C09. The region on A09 does not colocalize with QTL for seed weight detected by [28], and nor did a further region on A07, probably because [28] used a population spanning winter-type, semi-winter and spring-type *B.napus* representing highly divergent gene pools. All the same, the high subgenome homology between associated regions shows that seed yield in *B.napus* may indeed benefit from fixed heterosis among homoeologous alleles, as proposed by Basunanda and co-workers [51]. This is strengthened by the pyramiding allelic effects for yield (Fig. 7).

Despite the low overlap of candidate regions for yield with flowering and plant height, we detected one region on A09 which was conserved for all traits, containing *AGL* transcription factor *Bna.FUL* and histone methylation factor *Bna.GRP3*. While the former was detected in 2 copies for BOF-REL, the latter was detected in 3 copies for YIE-ABS. *FUL* was found to regulate not only flowering meristems, but all meristems [52] and has been shown to be regulated by *NTT*, leading to altered patterning of the silique [53].

Although not annotated as such, we also consider some flowering and clock genes found among the associated regions to be possible adaptation loci influencing yield. These include two copies each of *Bna.FUL, Bna.FPF1* and *Bna.PRR5*, and one copy each of *Bna.TFL1, Bna.FT* and *Bna.PRR1/TOC1*. Some of those genes have already been found to have an impact on seed yield: *Ath.FPF1* was proposed to have a further role in shade avoidance [54]. *Ath.TOC1* and *Ath.PRR5* are components of the circadian clock, which has been shown to influence overall plant fitness in allopolyploids [55]. *Bna.TFL1* and *Bna.FT* have already been shown to influence seed yield [45], whereas this was also the case for *Ath.FUL* [53]. Further evidence is provided by sequence data for all copies of *Bna.FT* in a subset of 121genotypes (Fig. 6), showing a significant yield reduction for the minor allele in two environments (Giessen and Gross-Gerau 2012), whereas the trend was similar when all environments were considered.

To find further candidates for adaptation, we performed a comparative analysis for relative values in all German environments. An additional copy of *Bna.FUL* was implicated in changes in all three traits, further suggesting that this gene may represent a general adaptation factor. Other genes which could be confirmed by COMP were *Bna.SPL1* and *Bna.SPL4*.

Promising candidates for adaption genes are the clock components *Bna.CCA1* and *Bna.TIC*. Both were detected in Gross-Gerau in 2011 in a region inversely affecting height and yield. As discussed before, a general influence of the circadian clock on plant fitness (e.g., through regulation of chlorophyll content and starch metabolism) was already shown for allopolyploids [55]. It was also interesting to note that a copy of *FT* (*BnaA07g25310D)* was associated with both plant height and yield in Rauischholzhausen in 2011. This corresponds to the findings for YIE-ABS, where we detected a further copy of *FT* (*BnaC04g14850D)* in a region associating with yield, underlined by independent sequence data, and with previous data suggesting that *B. napus FT* influences seed yield [45].

Interestingly, sequencing also revealed considerable functional variance for *Bna.CO.* Three SNPs obtained from resequencing a subset of 121 lines for *CO* copies were associated with flowering time. *Bna.CO* are strong candidates for flowering time determination [56, 57] and have been considered as a candidates in many QTL studies [11, 24–26], but were not detected in any region associated to flowering time in the present study. All the same, the very low LD between the flanking markers of two copies onA10 and C09 in particular suggests that strong selection might have broken down LD in this area. This highlights the importance of an improved reference sequence assembly, which will then allow anchoring of all important candidate genes to associated regions. Meanwhile, the presented data represent a valuable base for genetic studies and may assist in developing new breeding strategies for this important oil crop.

## Conclusions

We identified 101 *B.napus* genome regions associating with the onset of flowering, 69 with plant height and 36 with seed yield in highly diverse field environments. A total of 68 cross-trait regions were delineated. Generally, the pattern of associating regions was very environment specific, especially for flowering time, indicating a high influence of local field conditions. Several potential candidate genes for plant adaptation were found in chromosome regions associating with two or more traits. These include components of the circadian clock like *Bna.CCA1* and *Bna.TIC*, the flowering regulators *Bna.FT* and *Bna.FUL*, and developmental factors like *Bna.SPL4*. Using GO enrichment we found that trait-associated regions are enriched for genes involved in post-transcriptional regulation of gene expression, indicating that processes like RNA interference represent an important regulatory mechanism for flowering time determination in winter-type *B.napus* under field conditions. Whereas homologs of the basic flowering regulators *Bna.CO* and *Bna.FLC* where not detected in regions associated with flowering time, we found orthologs to several genes interacting either directly or indirectly with *SOC1*, an important signal integrator in the flowering pathway. Interestingly, AGL transcription factors were particularly strongly represented. Several flowering time regulators, including an homolog of *TFL1,* were detected in regions associated with seed yield. Our data suggest that numerous genes directly or indirectly involved in flowering regulation play pleiotropic roles in adaptation to environmental stresses, and thus represent important genetic factors to be considered for adaptive breeding. The interaction of post-transcriptional and epigenetic networks with adaptation genes and stress networks in contrast to classical transcription factors represents an important area for future research in this regard,. Regarding yield, pyramiding positive haplotypes detected in this study might assist in increasing yield further, but introgression of new genetic diversity into the winter oilseed rape breeding pool is necessary to stabilize yield gains in the face of environmental change.

## Methods

### Plant Material

The plant material used in the present study was selected from the ERANET-ASSYST *B.napus* diversity set, a panel of over 500 homozygous inbred lines (S5 or greater) described in [35, 36]. A panel of 158 adapted winter-type accessions were grown in different locations in Germany (Rauischholzhausen, Giessen, Gross Gerau) from 2009 until 2013, in southwest China (Beibei, Chongqing) from 2012 to 2013, and in central Chile (Temuco) in 2011 and 2012, as described in [34]. In all cases the plants were grown in autumn-sown trials and harvested the following spring or summer. In Germany, where winters generally have prolonged periods with temperatures well below freezing, the trials were sown in late August or early September, with harvest the following July. In southern-hemisphere Temuco, where the winter is comparatively short and temperatures are seldom below zero, the trials were sown in mid-April and harvested in January, while in the sub-tropical climate of Beibei winter-type rapeseed must be planted late September or early October to prevent flowering before winter. Although temperatures are then sufficiently cold to vernalise winter rapeseed, the cold period is very short so that flowering occurs much quicker than under northern European conditions.

### Field trials

Plots for phenotyping and DNA extraction were sown in a completely randomized block design with a harvest plot size of 3 x 1.25 m in 1 replicate (containing around 200 plants) except for Gross Gerau 2012, where 2 replicates were sown. Positional effects were controlled using an early flowering and a late flowering control genotype in 4 replicates, including them in the block design. When both genotypes were significantly separated using Student’s *t*-test on a *p* < 0.05 base, we concluded that the spatial impact was negligible. This was the case for all locations studied with the exception of GI2011 (*p* = 0.251). Single replication trials are standard procedure for determination of flowering time due to the high heritability. Plant protection and fertilisation was performed according to normal practice at the respective locations, as necessary for the prevailing conditions.

### DNA isolation

Leaf material for genomic DNA extraction was harvested from each accession in spring 2012 from the field trial in Giessen, Germany. Pooled leaf samples were taken from at least 5 different plants per genotype, immediately shock-frozen in liquid nitrogen and kept at −20 °C until extraction. Leaf material was ground in liquid nitrogen with a mortar and pestle. DNA was extracted using a common CTAB protocol modified from Doyle and Doyle (1990) as described earlier [34]. DNA concentration was determined using a Qubit fluorometer and the Qubit dsDNA BR assay kit (Life Technologies, Darmstadt, Germany) according to the manufacturer’s protocol. DNA quantity and purity was further checked on 0.5 % agarose gel (3 V/cm, 0.5xTBE, 120 min).

### Phenotyping

Flowering times were recorded for 158 accessions over a total of 11 environments from 2010–2012 at the 5 locations Giessen, Germany (50° 35′ N, 8° 40′ E), Gross Gerau, Germany (49° 55′ N, 8° 29′ E), Rauischholzhausen, Germany (50° 46′ N, 8° 53′ E), Beibei, Chongqing, China (29° 33′ N, 106° 34′ E) and Temuco, Chile (38° 44′ S, 72° 36′ W)). The onset of flowering was defined as the day where 10 % of the plants in a plot (3.75 m^2^) reached BBCH 61. Plant height in cm was measured at the onset of flowering. Plots were harvested in July (Germany) and January (Chile) and seed yield (corrected to dt/ha) was determined.

### SNP genotyping and data preprocessing

The 158 accessions were genotyped using the Brassica 60 K Illumina® Infinium SNP array by TraitGenetics GmbH (Gatersleben, Germany). Flanking sequences of 52,160 called SNPs were localized by BLAST to the *B.napus* Darmor-bzh reference genome assembly v4.1 [20], allowing no less than 50 bp overlap, 95 % sequence identity, and no gaps. SNPs with ambiguous position and SNPs on chr_random were excluded from the analysis, leaving 28,698 unique, locus-specific SNPs with a defined genome position. Heterozygous calls were treated as missing values. After preprocessing the marker set for non-missing marker values >0.9, minor allele frequency > 0.01 and non-missing individual markers >0.8 we retained 21,623 unique SNP markers and a population of 140 individuals for genome-wide association analysis. This resulted in a mean marker density of 1138 markers per chromosome, covering approximately 32.8 kbp/marker. Data preprocessing was performed with R (version 3.1.0) using the package GenABEL [58].

### Association analysis

Population structure analysis and visualization were performed in R (version 3.1.0) using the packages SelectionTools (http://fb09-pg-s207.agrar.uni-giessen.de/~frisch-m/), ape and scatterplot3d that apply Principal Component Analysis based on genetic distances calculated according to the euclidean modified Rodger’s distance method. The most likely number of population subclusters was estimated by plotting the within-cluster sum of squares against the possible number of clusters, ranging from 1 to 15 (see also Additional file 2: Figure S1). K-means clustering was then performed in R using SelectionTools. Genome-wide association testing and calculation of genome-wide allele identity-by-descent were performed with GenABEL. *F*_*ST*_-values were calculated with the program Genepop (version 4.2.2, http://kimura.univ-montp2.fr/~rousset/Genepop.htm) [59]. Linkage disequilibrium (LD) was calculated in terms of coefficient of determination (*r*^*2*^) in R, separately for each chromosome. Decay of LD was displayed using smoothed scatterplot curves with a span of 0.1.

Association analysis for flowering time was carried out using a mixed model with PC-adjustment using 21,623 unique SNPs for 11 environments, of which one was replicated. Using Bonferroni correction, only associations with *p* < 4.6*10-6 (giving a –log10(p) = 5.33) would prove significant on a *p* < 0.1 level. Predominantly weak associations are expected for highly quantitative traits like flowering time within an adapted gene pool. Because only 3 of the tested environments had SNPs meeting this condition, associations were considered as significant if –log10(p) > 4, as suggestive if –log10(p) > 3 and as supporting if –log10(p) > 2, based on FDR cutoff calculations performed with the R package fdrtool, proposing a cutoff of –log10(p) > 2. SNPs were considered for further analysis if either (1) a significant association was detected in at least one environment, or (2) at least one suggestive and one supporting association were detected in respective environments. Regions of interest were defined using the LD decay around the most significantly associated SNP markers, extending until r^2^ decayed to less than 0.5, meaning that regions extended from the left unlinked marker to the right unlinked marker. For plant height and yield, the analysis was performed likewise, with 10 and 8 environments, respectively. In a second approach, phenotypes were calculated as deviation from mean for every genotype in every location (see below), and association analysis was performed likewise.

$$ BOF{(rel)}_{envx}= mean\left(BOF(abs)\right)-BOF{(abs)}_{envx} $$

For comparative analysis in German environments, SNPs were considered as associated if they were at least suggestive for one trait and supporting for another.

Association analysis for the beginning of flowering (BOF) was performed in two ways: once using the absolute flowering time measured as days after sowing (considered as BOF-ABS), and once calculated as BOF-REL. This on the one hand allows us to find loci responsible for early or late flowering in the respective environment (BOF-ABS), and on the other hand to detect loci which are responsible for differential flowering behaviour between environments (BOF-REL). For plant height and yield, association analysis was performed exclusively using absolute values (HEI-ABS, YIE-ABS). Candidate genes were selected based on GO terms, considering the terms listed below.

**Table:** 

COMP		
BOF-ABS + BOF-REL	HEI-ABS	YIE-ABS
flower	photosynthesis	lipid
vernalization	mitosis	terpenoid
photoperiod	mitotic	biosynthetic process
circadian	cell growth	catabolic process
floral	development	gluconeogenesis
vegetative to reproductive	biosynth	glycolysis
vegetative phase change	catabolic	amino acid transport
pollen	metabolic	embryo development
carpel	tricarboxylic acid cycle	embryo sac development
sepal	glycolysis	
petal		

Furthermore, we carried out a comparative analysis between all three traits for German environments (COMP). For this, relative values were calculated only considering German environments, to find regions important for differential behaviour of two or more traits, indicating adaptive potential.

### Allelic effects

To assess the allelic effects for all associated regions for BOF-ABS, HEI-ABS and YIE-ABS, we determined the allelic state for each highest associating marker and genotype. We assigned a value to each marker, “0” for the reference allele and the value given by association analysis for the alternative allele. We counted the number of positive/early and negative/late alleles and tested for allelic effects using least significant distance tests in R using the package “agricolae”.

### GO enrichment analysis

GO terms of all genes detected among the defined regions of interest were extracted in R, using the package GOstats (http://bioconductor.org/biocLite.R ), separately for the tags BP, MF and CC. Each GO term has a unique identifier with a defined frequency in the *Brassica napus* genome annotations [20]. The enrichment of each GO term is then determined using a hypergeometrical test using a p-value cut-off of 0.05. Results were filtered for GO terms with a count of at least 10, and visualized using the web-based program REVIGO ([39]), using the following settings: database: Arabidopsis thaliana, similarity: 0.5 (small), semantic similarity measure: SimRel.

### Availability of supporting data

SNP data are deposited in dbsnp (http://www.ncbi.nlm.nih.gov/projects/SNP/) and are available as Supporting data (Additional file 6: Table S3).

## Additional files

Additional file 1: Table S1. Pearson’s correlation coefficient (above diagonal) and p-values (below diagonal) for all phenotypic traits used in this study. Grey fields indicate correlations for the same environment. The environments are abbreviated as follows: Germany: Giessen (GI), Gross Gerau (GG), Rauischholzhausen (RH); Chile: Temuco (TE); China: Beibei, Chongqing (BB), with their respective year of harvest. (XLSX 26 kb) Additional file 2: Figure S1. Results of population structure analysis in the association panel of 158 winter-type accessions, displaying 3 clusters as determined by k-means clustering. (A) and (B) show two PCA plots (component 1 and 2; component 2 and 3, respectively). (C) The best number of clusters was estimated by plotting the within-sum of squares against the possible number of clusters. (D) shows the distribution of the genome-wide allele identity-by-descent (IBD) for the total set, whereas (F) to (H) show the same for each cluster. (E) shows the *F*_*ST*_-values between clusters (white fields) and the mean IBD values for each cluster (grey fields). (PNG 132 kb) Additional file 3: Table S2. Candidate genes for associating regions for BOF-ABS, BOF-REL, HEI-ABS, YIE-ABS and COMP. Each gene has a unique identifier according to [20] and a short sequence description. The table names the highest associating marker in the defined region in its LD block with *r* ^*2*^ > 0.5 as well as the SNP marker most closely linked to the respective gene with its *r* ^*2*^ value to the highest associating SNP marker, and the closest markers distance from the gene. It also gives the value of the highest association among the respective region. The next columns show the strength of association in the respective environment; * stands for –log10(*p*) > 2, ** for -log10(*p*) > 3 and *** for –log10(*p*) > 4, and the last columns show the absolute effect of the highest associating marker on the respective trait. (XLSX 553 kb) Additional file 4: Figure S2. Boxplots showing (A) flowering time values and (B) plant height for genotypes carrying the specified number of positive and negative alleles for Gross-Gerau in 2010. (ZIP 213 kb) Additional file 5: Figure S3. Gene ontology scatterplot constructed with REVIGO in R [35] for all genes in the associated regions for the following traits. (A) relative values of flowering time in respect to genotypic mean (BOF-REL) (B) absolute values for plant height (HEI-ABS) (C) absolute values for seed yield (YIE-ABS) (D) relative values for flowering time, plant height and seed yield (COMP). The treemaps account for all GO terms with a count of min. 10., and were constructed using the Arabidopsis thaliana GO term database, using a similarity of 0.5, with SimRel as semantic similarity measure. Colours indicate the p-value of enrichment according to the legend. The size of each bubble reflects the count of each term among the enriched term list. Terms with a –log(*p*) > 1.5 are ascribed to their bubbles. (ZIP 870 kb) Additional file 6: Table S3. SNP positions and allelic states for all 21,623 SNPs used to calculate associations. The positions refer to the reference sequence, version v4.1, published by [20], which can be found on http://www.genoscope.cns.fr/brassicanapus/data/. (XLSX 851 kb)

## Abbreviations

AGL: Agamous-like
BBCH: Biologische Bundesanstalt, Bundessortenamt und CHemische Industrie
BOF-ABS: Mode of analysis: flowering time, absolute values
BOF-REL: Mode of analysis: flowering time, relative values
BP: Biological process
bp: Base pairs
CC: Cellular component
*CCA1*: *Circadian clock- associated 1*
COMP: Mode of analysis: comparative
*CRY2*: *Cryptochrome 2*
CTAB: Cetyltrimethylammoniumbromid
DAS: Days after sowing
DNA: Desoxyribonucleic acid
FDR: False discovery rate
*FLC*: *Flowering locus C*
*FPF1*: *Flowering Promoting Factor 1*
*FRI*: *Frigida*
*FRL*: *Frigida-like*
*FT*: *Flowering locus T*
*FUL*: *Fruitful*
*FWA*: *Flowering Wageningen*
*FYF*: *Forever young flower*
*GA2OX2*: *Gibberellin 2-oxidase 2*
*GA20OX1*: *Gibberellin 20-oxidase 1*
*GI*: *Gigantea*
*GRP3*: *Glycine-rich RNA-binding protein 3*
GO: Gene ontology
GWAS: Genome-wide association study
HEI-ABS: Mode of analysis: plant height, absolute values
IBD: Identity by descent
LD: Linkage disequilibrium
*LSH1*: *Light-dependent short hypocotyls*
MF: Molecular function
miRNA: Micro-RNA
ncRNA: Non-coding RNA
PCA: Principal Component Analysis
*PRR*: *Pseudo response regulator*
*PHYC*: *Phytochrome C*
QTL: Quantitative trait locus
*RLP19*: *Receptor like protein 19*
RNA: Ribonucleic acid
S5: 5^th^ selfing generation
SNP: Single nucleotide polymorphism
*SOC1*: *Suppressor of Overexpression of Co 1*
*SPL*: Squamosa-promotor binding-like proteins
*SRR1*: *Sensitivity to red light reduced 1*
TBE: Tris-borate - EDTA
*TEM*: *Tempranillo*
*TIC*: *Time for coffee*
*TOE2*: *Target of eat 2*
*VIN3*: *Vernalisation insensitive 3*
YIE-ABS: Mode of analysis: seed yield, absolute values
*ZTL*: *Zeitlupe*
