# Supplementary figures and images for: Diverse regulatory factors associate with flowering time and yield responses in winter-type Brassica napus
Source: BMC Genomics. 2015 Sep 29;16:737. doi: 10.1186/s12864-015-1950-1 (PMC4589123; doi:10.1186/s12864-015-1950-1)

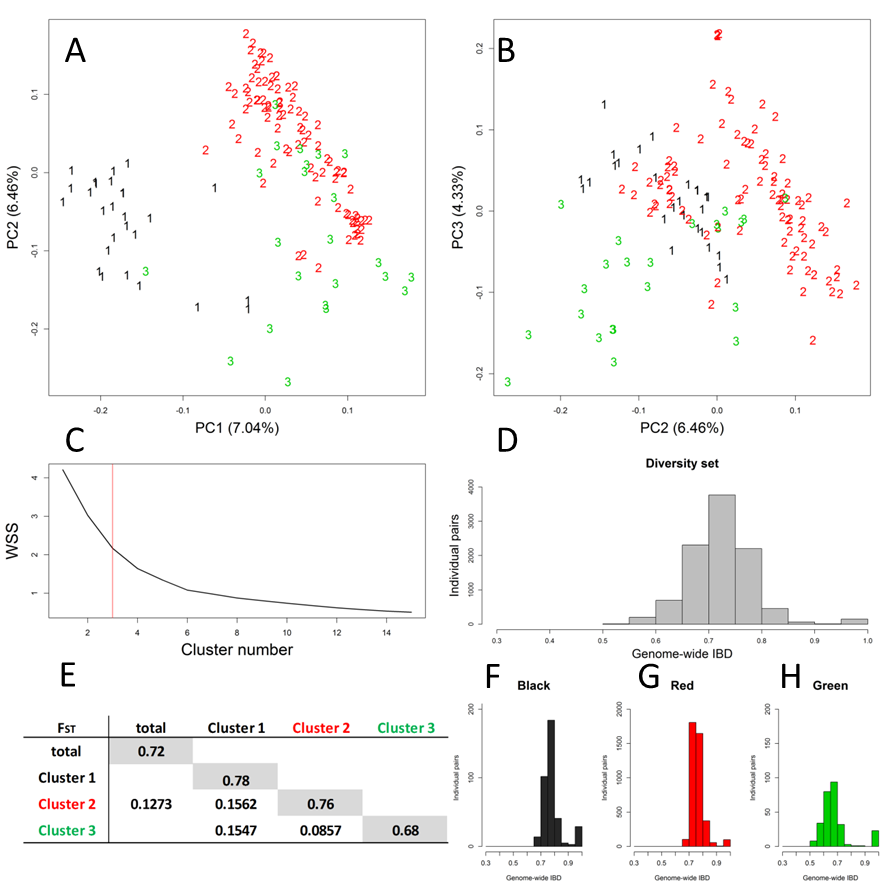

Supplement: Additional file 2: Figure S1. — Results of population structure analysis in the association panel of 158 winter-type accessions, displaying 3 clusters as determined by k-means clustering. (A) and (B) show two PCA plots (component 1 and 2; component 2 and 3, respectively). (C) The best number of clusters was estimated by plotting the within-sum of squares against the possible number of clusters. (D) shows the distribution of the genome-wide allele identity-by-descent (IBD) for the total set, whereas (F) to (H) show the same for each cluster. (E) shows the F ST-values between clusters (white fields) and the mean IBD values for each cluster (grey fields). (PNG 132 kb) [file 12864_2015_1950_MOESM2_ESM.png]

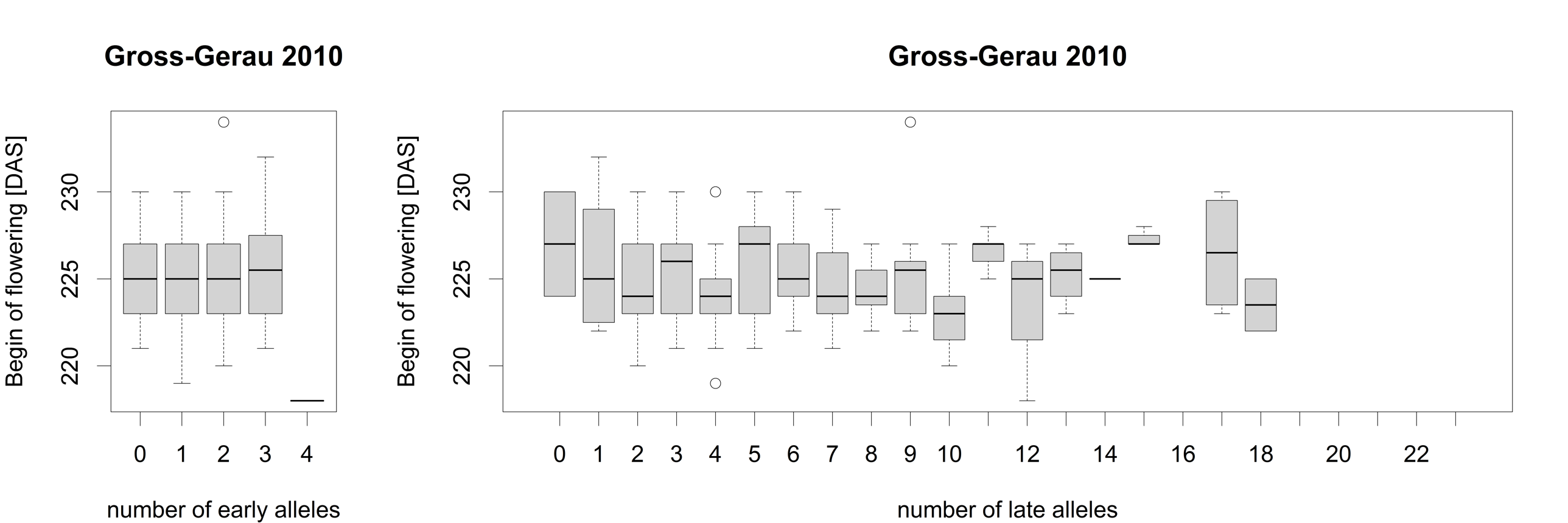

Supplement: Additional file 4: Figure S2. — Boxplots showing (A) flowering time values and (B) plant height for genotypes carrying the specified number of positive and negative alleles for Gross-Gerau in 2010. (ZIP 213 kb) [file 12864_2015_1950_MOESM4_ESM.zip › figure_s2A-B_revision/figure_s2A_revision.png]

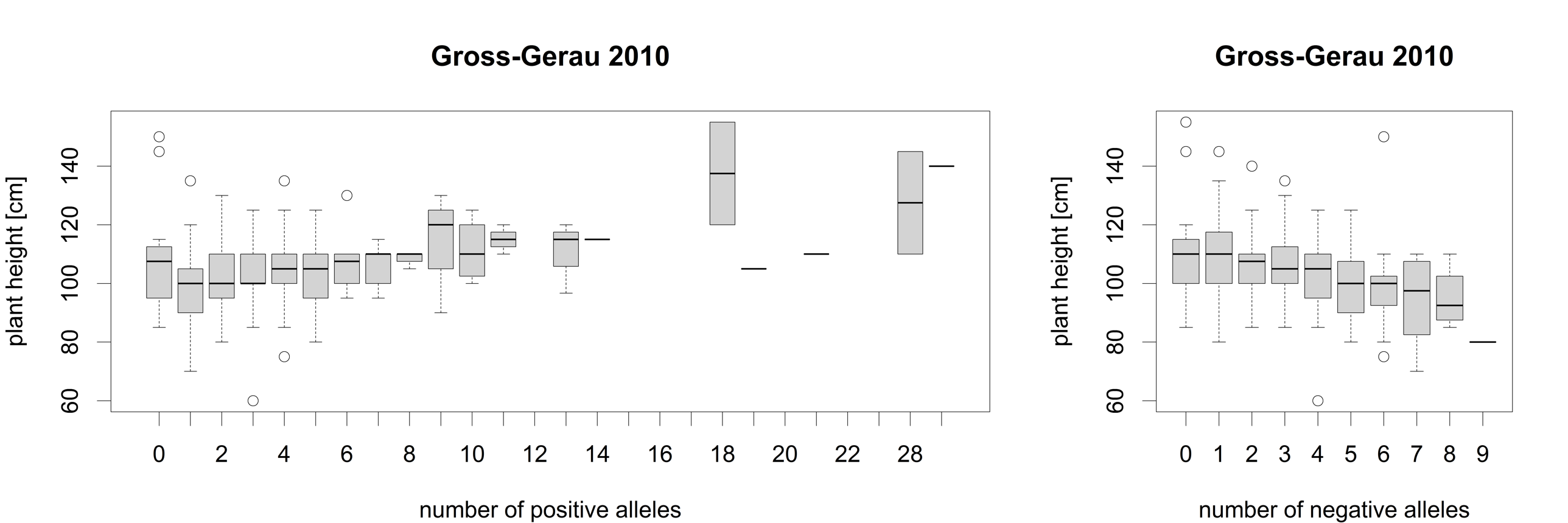

Supplement: Additional file 4: Figure S2. — Boxplots showing (A) flowering time values and (B) plant height for genotypes carrying the specified number of positive and negative alleles for Gross-Gerau in 2010. (ZIP 213 kb) [file 12864_2015_1950_MOESM4_ESM.zip › figure_s2A-B_revision/figure_s2B_revision.png]

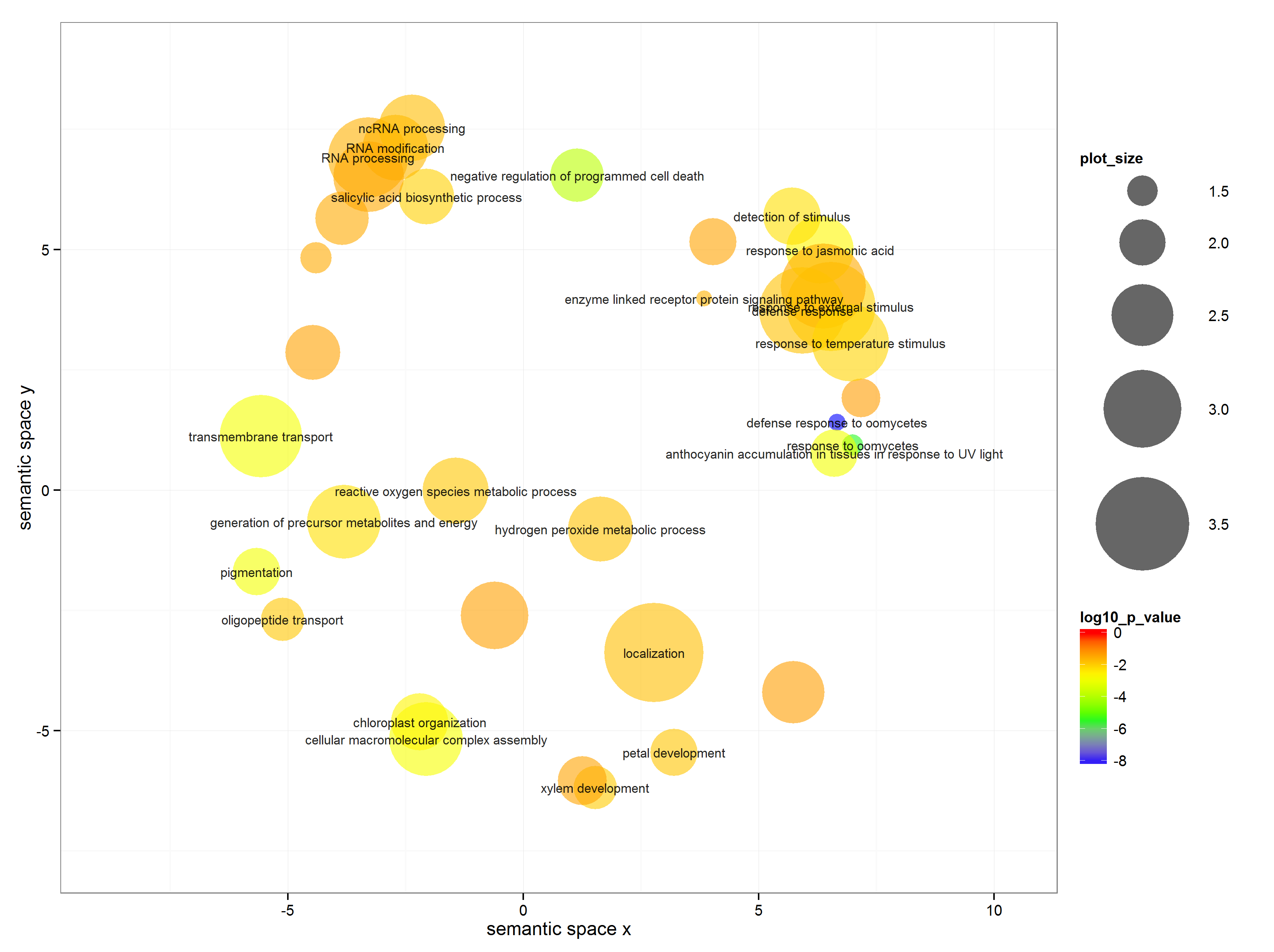

Supplement: Additional file 5: Figure S3. — Gene ontology scatterplot constructed with REVIGO in R [35] for all genes in the associated regions for the following traits. (A) relative values of flowering time in respect to genotypic mean (BOF-REL) (B) absolute values for plant height (HEI-ABS) (C) absolute values for seed yield (YIE-ABS) (D) relative values for flowering time, plant height and seed yield (COMP). The treemaps account for all GO terms with a count of min. 10., and were constructed using the Arabidopsis thaliana GO term database, using a similarity of 0.5, with SimRel as semantic similarity measure. Colours indicate the p-value of enrichment according to the legend. The size of each bubble reflects the count of each term among the enriched term list. Terms with a –log(p) > 1.5 are ascribed to their bubbles. (ZIP 870 kb) [file 12864_2015_1950_MOESM5_ESM.zip › figure_s3A-D_revision/figure_s3A_revision.png]

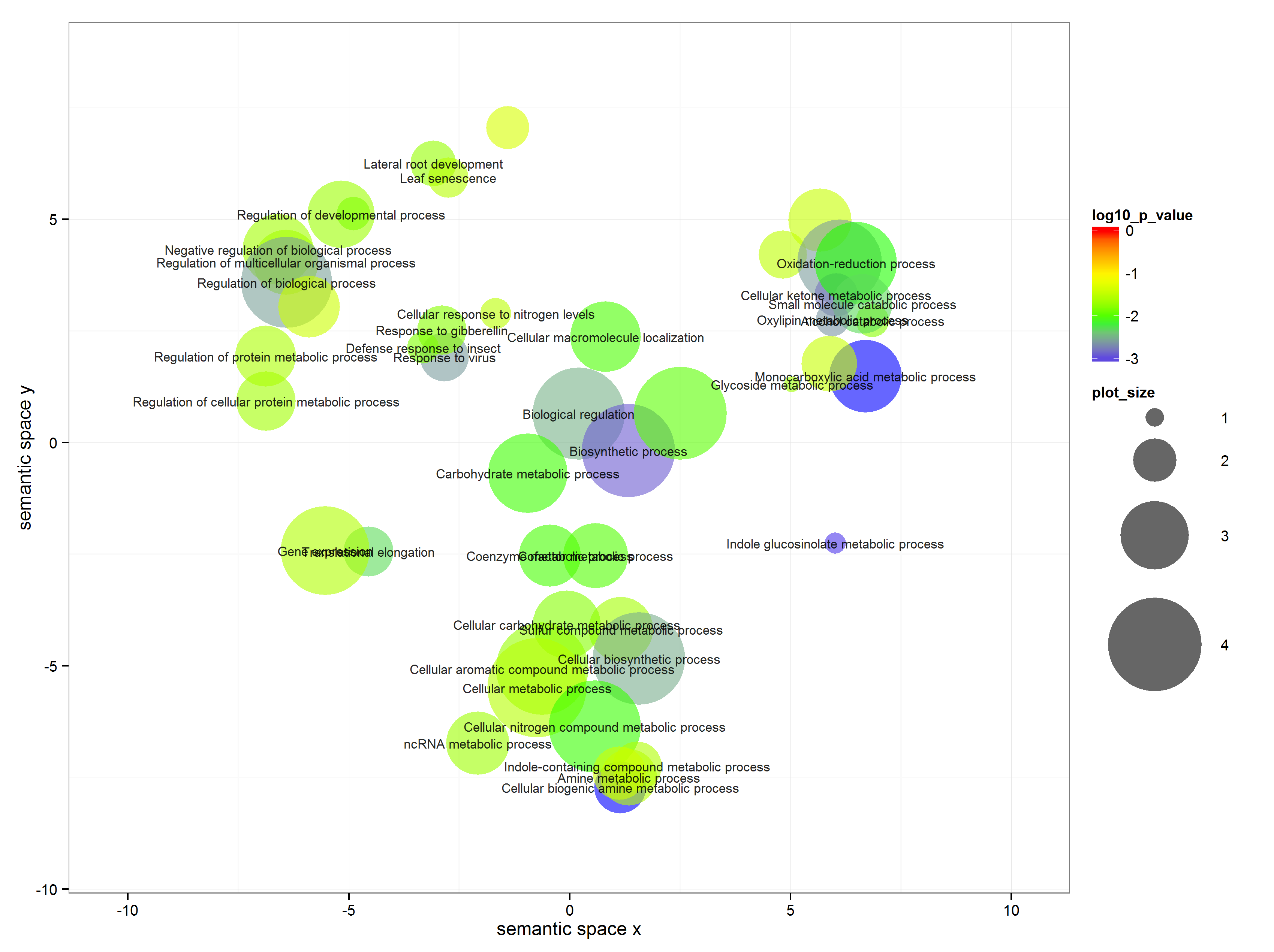

Supplement: Additional file 5: Figure S3. — Gene ontology scatterplot constructed with REVIGO in R [35] for all genes in the associated regions for the following traits. (A) relative values of flowering time in respect to genotypic mean (BOF-REL) (B) absolute values for plant height (HEI-ABS) (C) absolute values for seed yield (YIE-ABS) (D) relative values for flowering time, plant height and seed yield (COMP). The treemaps account for all GO terms with a count of min. 10., and were constructed using the Arabidopsis thaliana GO term database, using a similarity of 0.5, with SimRel as semantic similarity measure. Colours indicate the p-value of enrichment according to the legend. The size of each bubble reflects the count of each term among the enriched term list. Terms with a –log(p) > 1.5 are ascribed to their bubbles. (ZIP 870 kb) [file 12864_2015_1950_MOESM5_ESM.zip › figure_s3A-D_revision/figure_s3B_revision.png]

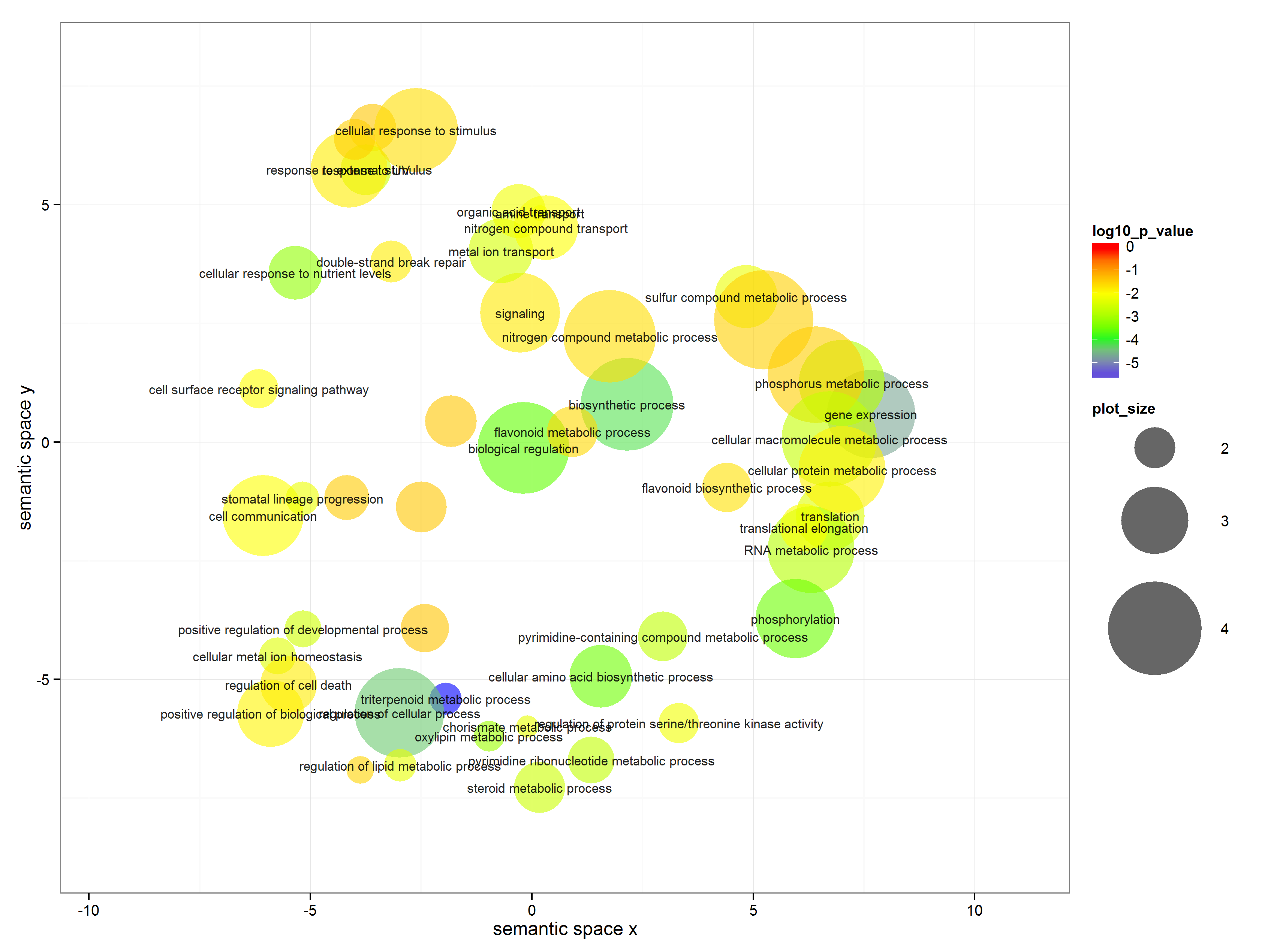

Supplement: Additional file 5: Figure S3. — Gene ontology scatterplot constructed with REVIGO in R [35] for all genes in the associated regions for the following traits. (A) relative values of flowering time in respect to genotypic mean (BOF-REL) (B) absolute values for plant height (HEI-ABS) (C) absolute values for seed yield (YIE-ABS) (D) relative values for flowering time, plant height and seed yield (COMP). The treemaps account for all GO terms with a count of min. 10., and were constructed using the Arabidopsis thaliana GO term database, using a similarity of 0.5, with SimRel as semantic similarity measure. Colours indicate the p-value of enrichment according to the legend. The size of each bubble reflects the count of each term among the enriched term list. Terms with a –log(p) > 1.5 are ascribed to their bubbles. (ZIP 870 kb) [file 12864_2015_1950_MOESM5_ESM.zip › figure_s3A-D_revision/figure_s3C_revision.png]

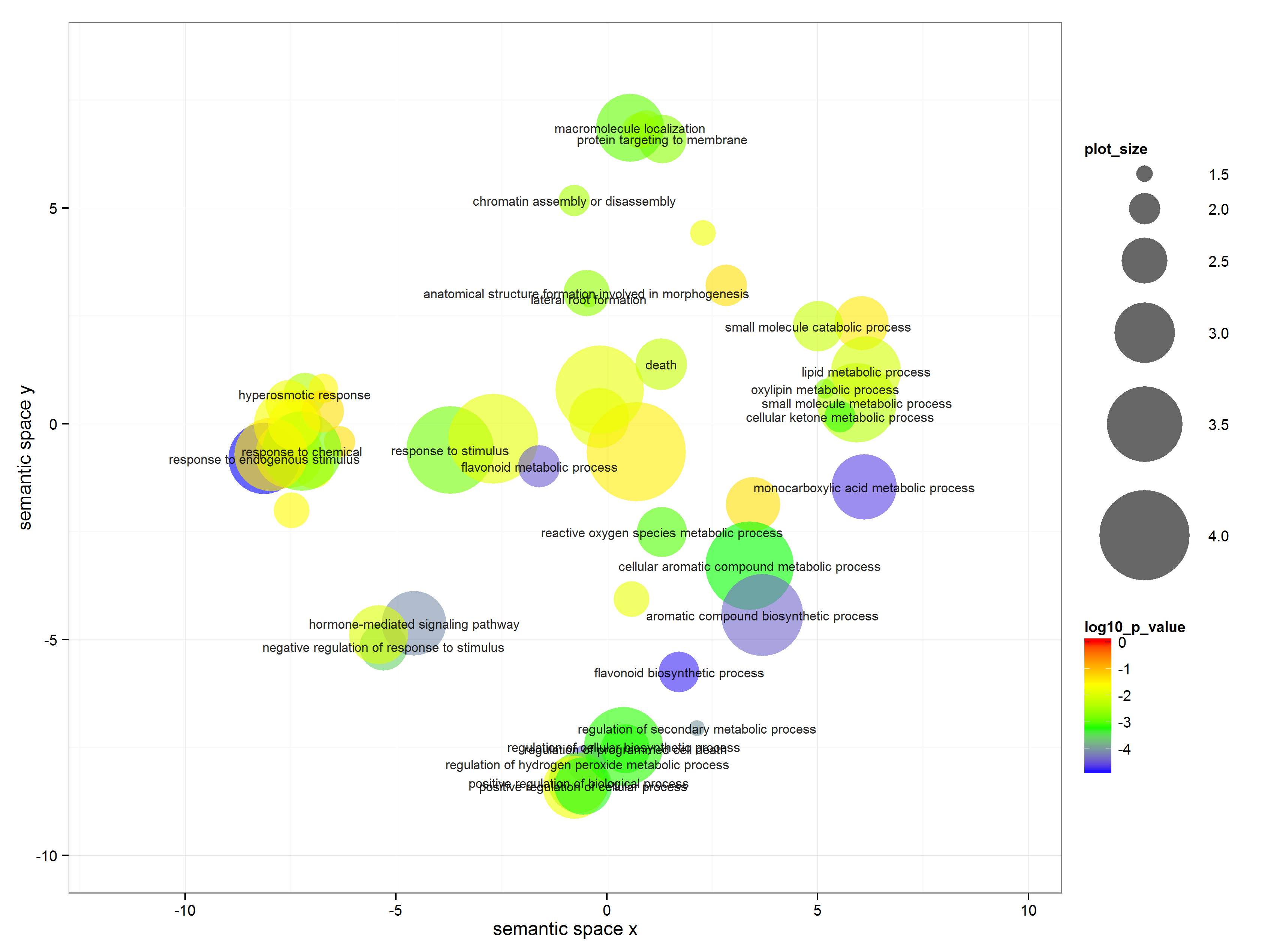

Supplement: Additional file 5: Figure S3. — Gene ontology scatterplot constructed with REVIGO in R [35] for all genes in the associated regions for the following traits. (A) relative values of flowering time in respect to genotypic mean (BOF-REL) (B) absolute values for plant height (HEI-ABS) (C) absolute values for seed yield (YIE-ABS) (D) relative values for flowering time, plant height and seed yield (COMP). The treemaps account for all GO terms with a count of min. 10., and were constructed using the Arabidopsis thaliana GO term database, using a similarity of 0.5, with SimRel as semantic similarity measure. Colours indicate the p-value of enrichment according to the legend. The size of each bubble reflects the count of each term among the enriched term list. Terms with a –log(p) > 1.5 are ascribed to their bubbles. (ZIP 870 kb) [file 12864_2015_1950_MOESM5_ESM.zip › figure_s3A-D_revision/figure_s3D_revision.png]
